# Graded Smad2/3 Activation Is Converted Directly into Levels of Target Gene Expression in Embryonic Stem Cells

**DOI:** 10.1371/journal.pone.0004268

**Published:** 2009-01-27

**Authors:** Marcela Guzman-Ayala, Kian Leong Lee, Konstantinos J. Mavrakis, Paraskevi Goggolidou, Dominic P. Norris, Vasso Episkopou

**Affiliations:** 1 Mammalian Neurogenesis, MRC Clinical Sciences Centre, Imperial College School of Medicine, Hammersmith Hospital, London, United Kingdom; 2 Mammalian Genetics Unit, MRC, Harwell, United Kingdom; Columbia University, United States of America

## Abstract

The Transforming Growth Factor (TGF) β signalling family includes morphogens, such as Nodal and Activin, with important functions in vertebrate development. The concentration of the morphogen is critical for fate decisions in the responding cells. Smad2 and Smad3 are effectors of the Nodal/Activin branch of TGFβ signalling: they are activated by receptors, enter the nucleus and directly transcribe target genes. However, there have been no studies correlating levels of Smad2/3 activation with expression patterns of endogenous target genes in a developmental context over time. We used mouse Embryonic Stem (ES) cells to create a system whereby levels of activated Smad2/3 can be manipulated by an inducible constitutively active receptor (Alk4*) and an inhibitor (SB-431542) that blocks specifically Smad2/3 activation. The transcriptional responses were analysed by microarrays at different time points during activation and repression. We identified several genes that follow faithfully and reproducibly the Smad2/3 activation profile. Twenty-seven of these were novel and expressed in the early embryo downstream of Smad2/3 signalling. As they responded to Smad2/3 activation in the absence of protein synthesis, they were considered direct. These immediate responsive genes included negative intracellular feedback factors, like SnoN and I-Smad7, which inhibit the transcriptional activity of Smad2/3. However, their activation did not lead to subsequent repression of target genes over time, suggesting that this type of feedback is inefficient in ES cells or it is counteracted by mechanisms such as ubiquitin-mediated degradation by Arkadia. Here we present an ES cell system along with a database containing the expression profile of thousands of genes downstream of Smad2/3 activation patterns, in the presence or absence of protein synthesis. Furthermore, we identify primary target genes that follow proportionately and with high sensitivity changes in Smad2/3 levels over 15–30 hours. The above system and resource provide tools to study morphogen function in development.

## Introduction

TGFβ signalling controls a diverse set of cellular processes including cell proliferation, differentiation, apoptosis, and specification of developmental fate in vertebrate and invertebrate species. Disruption of signalling leads to developmental abnormalities and disease, including cancer. TGFβ comprise a large family of secreted factors that bind to pairs of membrane receptor serine/threonine kinases (receptor types I and II), which then phosphorylate the Smad effectors at their C terminus (P-Smad), allowing them to complex with the common factor Smad4 leading to nuclear translocation [1]–[4]. There are two signalling branches: One of these includes morphogens like Nodal and Activin, which activate the Smad2 and Smad3 (Smad2/3) effectors [4]. P-Smads bind to DNA directly and/or interact with different DNA-binding partner cofactors such as FoxH1, which bind to specific enhancers and confer target gene specificity [5]. It is estimated that hundreds of genes are regulated directly by Smad2/3, most of which are activated, although some are repressed [5], [6]. Several Smad target genes have been identified during development but only a few have been shown to be direct [7]–[9].

The divergent functions of TGFβ ligands critically depend on the concentration to which the responding cell is exposed. Studies of morphogen gradients have shown that Nodal is a key TGFβ morphogen in vertebrate development responsible for gastrulation, germ layer formation and patterning, i.e. shaping the embryo by specifying the axes of the body plan [10]. Therefore, the multiple functions of Nodal depend on concentration and exposure of cells to different levels activates specific genes and distinct cell fates [11], [12]. Loss of function mutations in the *Nodal* gene, including deletions of regulatory elements that, lead to a reduction of *Nodal* levels of expression [13], reveal that the highest level of Nodal signalling is required during gastrulation for the induction of the anterior primitive streak, which gives rise to the mammalian equivalent to Spemann's organiser. Complementary experiments in *Xenopus* embryos, show that increasing amounts of *Nodal* RNA injection into naïve cells, induces different cell fates at a dose-dependent manner, and that the highest level induces Spemann's organiser [14]. How signalling levels elicit specific transcriptional responses within the cell remains elusive. In cell-line transcriptional assays with reporter constructs driven by target gene promoters, the levels of the activated Smad2/3 (P-Smad2/3) reflect signalling intensity (ligand activated receptors) and these are proportionate to the levels of reporter expression. However, correlation of P-Smad2/3 levels with expression patterns of endogenous target genes over time during development had not been examined.

To efficiently manipulate activation of Smad2/3 in a cellular environment relevant to embryonic development, and where Nodal/Activin are known to function as morphogens, we used ES cells. ES cells are pluripotent cells derived from the inner cell mass of blastocysts. They can self renew in culture indefinitely without losing their normal karyotype and their ability to differentiate [15]. When they are introduced back to host blastocysts they contribute to all cells of the embryo including the germ line, indicating their pluripotent stem cell identity [16], [17]. In addition, ES cells can be manipulated to differentiate in culture and they therefore present an excellent embryonic system for studying molecular and cellular aspects of cell fate and differentiation [18]. Mouse ES cells exhibit high levels of autocrine Smad2/3 signalling and express several TGFβ signalling factors including Nodal [19]. It is difficult to manipulate signalling as only a weak enhancement of Smad2/3 activation can be achieved by addition of Activin in the medium [19]. Furthermore, Nodal/Activin treatment of ES cells has diverse effects: from maintenance of proliferation and pluripotency of human ES cells [20]–[22], to their differentiation towards endoderm [23]–[26]; a lineage known to depend on robust levels of Nodal signalling during vertebrate embryogenesis [27]. The mechanism by which ES cells respond to Nodal/Activin in very different ways remains unknown. However, levels of Nodal/Activin seem to be critical for the specific outcome [28].

The intracellular levels of P-Smad2/3 are influenced by the abundance of receptors, extracellular co-receptors and antagonists, all of which control the exposure of cells to the ligand. Several of the genes encoding extracellular and intracellular regulators are themselves direct downstream targets of Smad2/3 activity (feedback mechanisms) [6]. In addition, while Activin can bind and activate the receptors directly, Nodal requires the co-receptor Cripto [29]. As availability of Cripto determines the activity of Nodal but not that of Activin, it is impossible to predict the levels of the activated effectors within the cell, based on levels of extracellular ligand. Furthermore, the transcriptional activity of P-Smad2/3 is modulated by intracellular feedback mechanisms including co-activators and co-repressors [5], [30] and therefore, P-Smad2/3 levels do not always correspond to efficient target gene expression [19]. It is therefore unknown whether the transcriptional responses downstream of Nodal/Activin follow Smad2/3 activation levels and how the intracellular feedback mechanisms shape expression patterns over time.

To address how target genes respond to the concentration of P-Smad2/3, and to bypass the extracellular environment, we placed the activation of Smad2/3 under an inducible constitutively active receptor (Alk4*). This receptor is induced by a tetracycline analogue compound, Doxycycline (Dox), and can phosphorylate Smad2/3 in the absence of TGFβ ligands or other receptors. Furthermore, to block activation we used the specific inhibitor SB-431542, which targets the receptors (including the exogenous Alk4*) responsible for Smad2/3 phosphorylation [31]. We used this system to regulate the levels of Smad2/3 activation in a time course of induction and inhibition. We evaluated the activation by western blot and studied gene expression by microarrays at successive time points. We screened for genes that followed the Smad2/3 activation patterns. We then examined their expression during induction over time in the absence of protein synthesis and found twenty-seven novel genes to be upregulated; these genes were therefore considered to be immediate early primary targets. Semi-quantitative and quantitative PCR confirmed the expression patterns of these genes during induction/inhibition. Sequence analysis revealed the presence of conserved FoxH1/Smad2 binding elements [32], [33] in several of these genes supporting that they are direct targets. Furthermore, we showed that these genes are expressed in early stage mouse embryos and that their expression depends on Nodal signalling suggesting that they are relevant to development.

Our findings reveal that Smad2/3 activation levels are converted proportionately to transcriptional responses in ES cells and probably in early embryos. In addition to the novel target genes, regulatory factors (positive and negative) of the TGFβ signalling pathway were among the most readily responsive and direct target genes (feedback factors). However, the activation of negative intracellular feedback regulators, which interfere with P-Smad2/3 transcriptional activity, do not appear to have a major effect, as target gene expression remains sensitive and adjusts quickly to changes of Smad2/3 activation levels for at least a period of 15–30 hours. Our ES cell system along with the database containing the expression profile of thousands of genes in response to changing Smad2/3 activation over time, provide unique tools for a broad spectrum of scientists and studies. Understanding how the different functions of TGFβ factors are implemented will provide useful insights for morphogen function in development, stem cell maintenance and differentiation, as well as diseases such as cancer.

## Results

### Efficient manipulation of Smad2/3 activation in TAG1 ES cells

**Figure 1:**
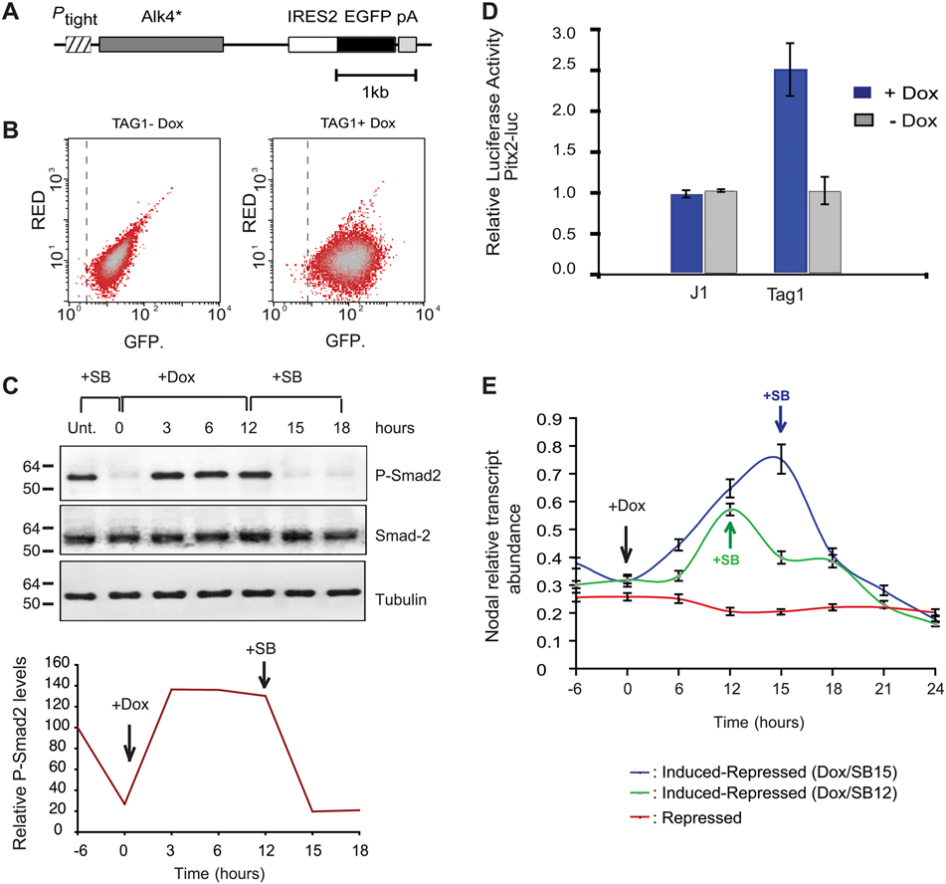
A tetracycline/Dox inducible Alk4*-receptor phosphorylates Smad2/3 and activates endogenous and exogenous targets in ES cells. (A) Schematic representation of the Alk4* inducible expression construct *pSLTT-AIG*. The tet-on inducible promoter (*P*_tight_) drives the transcription of a bicistronic message encoding the constitutive active Alk4* receptor (dark grey bar) followed by an internal ribosome entry site (IRES2, open bar), a GFP reporter (EGFP, black bar) and a polyadenylation signal (pA, light grey bar). The scale bar represents 1kb. (B) Two-colour FACS analysis of stable TAG1 ES cells cultured in the presence or absence of Dox for 18 hours. The y-axis represents red fluorescence while the x-axis represents GFP (green) fluorescence, both on a log_10_ scale. Over 90% (broken line) of the Dox induced (TAG1+Dox) ES cells display specific green fluorescence compared to the uninduced (TAG1-Dox) cells. (C) Western blot analysis of P-Smad2 levels during Alk4* induction and subsequent SB inhibition in TAG1 ES cells. Cell extracts of each time point were analysed with P-Smad2, Smad2 and Tubulin (loading control) antibodies. The curve derives from the densitometry analysis of the P-Smad2 bands on the western blot normalised against the Tubulin bands. All values are expressed relative to the untreated control (Unt.) represented as 100%. (D) Luciferase assays of the parental J1 cell line (cells that do not contain *pSLTT-AIG* construct) and TAG1 ES transfected with the *Pitx2* luciferase reporter construct. Bars represent the relative increase of luciferase activity in the induced cells (blue) compared to uninduced cells (grey), for both J1 and TAG1 ES cells. Error bars represent the standard deviation in biological triplicates (n = 3). (E) Quantitative real-time PCR of *Nodal* transcript in TAG1 ES cells treated sequentially with Dox inducer and SB inhibitor (at 15 hours in blue or 12 hours in green). A control set of cells was kept for the duration of the experiment in SB (red line). Relative *Nodal* transcript abundance is expressed as the average of 4 PCR reactions (n = 4) normalised to the average of the housekeeping control genes *Gapdh*, *Ube*, *Ywhaz* and *B2m* with standard error of the mean of PCR reactions. Cells were pre-treated with SB for 6 hours to reduce autocrine-signalling and target gene expression (time point 0) in (C) and (E).

To address how target genes respond to the concentration of P-Smad2/3, we bypassed the extracellular environment by placing Smad2/3 activation under the control of a constitutively active Alk4 receptor (Alk4*), which can phosphorylate Smad2/3 in the absence of TGFβ ligands or other receptors [34]. In addition, we placed the expression of Alk4* under a tetracycline transactivator responsive promoter [35]. To turn off Smad2/3 phosphorylation we used the specific inhibitor SB-431542 (SB) [31], which blocks the TGFβ receptors Alk4/5/7 including the exogenous Alk4*, and is tolerated well by the ES cells [19]. A green fluorescent protein (GFP) reporter with an internal ribosomal entry site (IRES) was placed downstream of the Alk4* open reading frame to allow evaluation of transcription levels (Figure 1A). The inducible Alk4* construct (pSLTT-AIG) was stably integrated into the J1 R26/N-NLSrtTA (J1) ES cell line [36]. The J1 cell line contains the tetracycline dependent transactivator (rtTA) stably integrated via homologous recombination into the ubiquitously expressed ROSA26 locus. The tetracycline analogue Dox, was used to activate the rtTA-induced transcription of Alk4*. An ES cell clone, TAG1, showed high expression of GFP after Dox treatment (Figure 1B), and therefore, was selected for further experiments. As Alk4* precedes GFP in the dicistronic construct, it is expected to be expressed in TAG1 cells.

Alk4* expression and function were tested by analysing the activation of Smad2 (P-Smad2) after Dox or SB treatment in western blots. Dox treatment resulted in an upregulation of P-Smad2 by 2.6-fold after 12 hours, which was maintained up to 24 hours (Figure S1A). No significant changes in total Smad2 levels were observed (Figure S1A). We then followed Smad2 activation (by Dox) and inhibition (by SB) in a time course experiment (Figure 1C). We turned off the autocrine-signalling present in ES cells, with SB for 6 hours (time point zero; 0) prior to Dox treatment (Alk4* induction); this pre-treatment was used in all subsequent time course experiments. During induction of Alk4* (with Dox), P-Smad2 levels were increased (>5-fold) after 3 hours compared to time point 0 (T0). As we did not observe any further increase after 6 or 12 hours, we assumed that activation of Smad2/3 becomes saturated at this early time point. In the second phase of the experiment (inhibition), addition of SB inhibitor after 12 hours of induction caused a rapid reduction of P-Smad2 levels (>5 fold) within the first 3 hours (time point 15). Collectively the above data show that in TAG1 cells Dox induces functional Alk4*, which can also be blocked by SB. We have therefore developed a system to efficiently manipulate the receptor-dependent phosphorylation of Smad2/3 in ES cells.

### Exogenous and endogenous target gene expression follows the Smad2/3 activation profile in ES cells

We examined the transcriptional activity of the Alk4* induced P-Smad2/3 in the TAG1 system using an exogenous luciferase reporter 0.9-P1, which is driven by the Smad2 regulated promoter of *Pitx2* gene [19] (Figure 1D) in transient transfection assays. The reporter activity was increased 2.2-fold in TAG1 ES cells treated with Dox compared to the untreated control, while no difference was observed in the parental J1 cells, which contain only the rtTA and not the inducible Alk4* (Figure 1D).

We also examined the endogenous P-Smad2/3 transcriptional response downstream of the Dox-induced Alk4* in TAG1 cells by examining with quantitative reverse transcription PCR (RT-PCR) the expression profile of the known target gene *Nodal* over time (Figure S1B). We found that *Nodal* expression was upregulated 1.5-fold within the first 6 hours of activation and 2.5-fold after 12 hours (green trend line in Figure S1B). In untreated cells, *Nodal* transcription was increased upon removal of SB after pre-treatment at time point T0 due to de-repression of the autocrine signalling (blue trend line in Figure S1B). *Nodal* expression did not increase in the cells that SB was maintained throughout the experiment (red line in Figure S1B). Therefore Nodal, and possibly other endogenous target genes, respond to the activation of Smad2/3 downstream Alk4* induction.

The TAG1 cells were then used to perform a two-phase signalling, Dox/SB, time course experiment. Following 6 hours of SB pre-treatment, the cells were maintained in Dox for 15 hours and subsequently treated with SB for 9 hours (Dox/SB15). We collected mRNA at different time points and analysed *Nodal* expression by quantitative RT-PCR (Figure 1E). We repeated the two-phase experiment to examine the reproducibility of the *Nodal* transcriptional responses. In addition, to examine the sensitivity of the target genes in response to SB treatment, and to see whether negative feedback mechanisms interfered with their response at 15 hours, in the repeated experiment the inhibitor was added 3 hours earlier (at 12 hours; Dox/SB12). We observed similar response of *Nodal* expression in both experiments (Figure 1E; Dox/SB15 blue and Dox/SB12 green curve). *Nodal* expression increased by 1.5-fold in the first 6 hours of induction and peaked at 2.5 fold after 15 hours, while it was reduced by almost 2-fold in the first 3 hours of inhibition (time point 18) and in 6 hours it returned to basal levels of expression (time point 21). The fast downregulation of *Nodal* upon SB treatment after 12 or 15 hours indicates that there are no secondary mechanisms activated by Smad2/3 over time and that the Nodal promoter remains sensitive to changes of Smad2/3 activation in ES cells. In TAG1 cells cultured continuously (30 hours) with SB inhibitor (red trend line in Figure 1E), *Nodal* levels were maintained at basal levels indicating that Smad2/3 activation is responsible for the upregulation of *Nodal* under Dox/Alk4* induction. Collectively the above data show that the manipulation of Alk4*-Smad2/3 activation in TAG1 ES cells elicit downstream transcriptional responses on both exogenous and endogenous promoters.

### Sixty genes followed directly the pattern of Smad2/3 activation in TAG1 ES cells

We screened for additional P-Smad2/3 target genes that follow the expression profile of *Nodal* using microarrays (Affymetrix) on the mRNA samples collected from the various time points in two experiments described above, Dox/SB15 and Dox/SB12 (Figure 1E). We considered as target genes those that meet the following criteria: upregulation by ≥1.2 fold in the second or third time point during induction; downregulation by ≥1.2 fold at least in one of the time points during inhibition; genes were not included if one of the set probes did not behave similarly in both experiments; or the “p” values were not statistical significant (p>0.01) at any of the time points.

In total, 64 genes satisfied the criteria in both experiments (Table 1, Table S1 and Table S2). This list of genes included known Smad2/3 target genes such as *Nodal*, *Pitx2*, *Lefty1* and *Lefty2* [33], [37], which indicate that our criteria were appropriate for the identification of targets. The Alk4 receptor (Acvr1) was also found in the list; however, it cannot be distinguished from the exogenous Alk4*, which is upregulated by Dox/rtTA, and therefore, cannot be included in our list of Smad2/3 targets.

**Table 1:** Behaviour and classification of gene expression in the Dox/SB15 experiment.

High Response Targets (10- to 100-fold)					
			Tag1 Cells		J1 cells
Gene	Probe Sets ID	Accession	0h–15h (Dox)	0h–21h (Dox/SB)	0h–12h (Dox)
Pitx2	1424797_a_at	U80011	100	29.398	1.572
Lefty1	1417638_at	NM_010094	51.985	6.470	2.215
GalNAcS-6ST	1452092_at	AK019474	36.318	5.456	7.34
Lefty2	1436227_at	AV214969	22.692	5.450	−1.008
Pitx2	1450482_a_at	AB006320	20.173	4.568	1.519
Fgf15	1418376_at	NM_008003	15.146	7.666	3.16

Medium Response Targets (2.5- to 10-fold)					
			Tag1 Cells		J1 cells
Gene	Probe Sets ID	Accession	0h–15h (Dox)	0h–21h (Dox/SB)	0h–12h (Dox)
1443256_at	1443256_at	BB548833	8.196	5.175	2.237
Pcdh8	1447825_x_at	BB076893	7.615	4.729	1.762
AW548124	1454838_s_at	BB323985	7.440	2.136	−1.139
Duxbl	1445710_x_at	AV321065	5.521	4.768	3.497
Smad7	1423389_at	BF226166	4.977	1.848	−1.277
Tmem63a	1423871_at	BC019442	4.956	1.866	1.112
Smad7	1443771_x_at	BB241324	4.792	1.771	−1.133
AW548124	1460411_s_at	BC022157	4.593	1.696	−1.078
Tmepai	1422706_at	AV370981	4.066	−1.040	−1.058
Cnpy1	1437996_s_at	BB131676	3.944	2.118	1.74
Nxn	1422465_a_at	BB366804	3.674	3.094	1.689
Bcar3	1415936_at	NM_013867	3.315	2.352	1.454
Pycr2	1448315_a_at	NM_133705	3.314	1.782	−1.034
Cd97	1418394_a_at	NM_011925	3.044	1.714	1.48
Plekha2	1417288_at	NM_031257	2.883	1.695	1.035
Slc7a7	1447181_s_at	AI790233	2.727	2.362	1.552
Lgr4	1433891_at	BI107632	2.625	1.637	1.171
Camk2n1	1456609_at	BE994488	2.583	1.601	1.356
Slc7a7	1417392_a_at	NM_011405	2.578	2.243	1.565

Low Response Targets (1.2- to 2.5-fold)					
			Tag1 Cells		J1 cells
Gene	Probe Sets ID	Accession	0h–15h (Dox)	0h–21h (Dox/SB)	0h–12h (Dox)
Abcg2	1422906_at	NM_011920	2.386	1.597	1.223
SnoN	1422054_a_at	U36203	2.355	2.227	1.855
Rasd2	1427344_s_at	BC026377	2.331	1.152	−1.091
Dusp9	1433845_x_at	AV295798	2.316	2.058	1.306
Bhlhb8	1449233_at	BC011486	2.292	1.897	1.966
Sntb2	1449840_at	BI646094	2.260	1.285	−1.063
Dusp9	1454737_at	AV295798	2.236	1.770	1.054
Nphs1	1422142_at	AF172256	2.225	1.744	1.634
Nodal	1422057_at	X70514	2.138	−1.243	1.088
SnoN	1452214_at	AK018608	2.041	1.927	1.532
Notch3	1421965_s_at	NM_008716	2.027	1.675	−1.043
Tmepai	1422705_at	AV370981	1.995	−1.150	1.235
D6Wsu176e	1417953_at	AK016470	1.994	1.551	1.28
Ubr7	1433479_at	AV030071	1.923	1.472	1.188
Pea15	1416407_at	AI323543	1.908	1.449	1.227
Ubr7	1454616_at	AV030071	1.863	1.375	1.436
Cripto	1450989_at	AV294613	1.841	1.706	−1.372
Bbc3	1423315_at	AW489168	1.826	1.073	−1.043
Gpr107	1459788_at	BB115649	1.761	−1.010	−1.874
Schip1	1423025_a_at	NM_013928	1.761	1.349	−1.011
Epha2	1421151_a_at	NM_010139	1.725	1.405	−1.405
Ppp1r2	1417341_a_at	NM_025800	1.651	1.382	1.185
Rhob	1449110_at	BC018275	1.636	1.224	−1.295
Ski	1429192_at	AV381512	1.625	1.214	−1.047
Zcchc11	1437395_at	BE370775	1.596	1.355	1.137
Atrx	1420948_s_at	BB825830	1.562	1.206	1.087
Mcl1	1416881_at	BC003839	1.549	1.308	1.046
Zfp423	1419380_at	NM_033327	1.535	1.032	1.135
Ccnd2	1416122_at	NM_009829	1.501	−1.582	1.064
Eif3s6ip	1437948_x_at	BB443362	1.499	−1.067	−1.386
Ccnd2	1434745_at	BQ175880	1.438	−1.524	1.141
5730419I09Rik	1437003_at	BB323930	1.424	−1.018	1.08
Aasdhppt	1428757_at	AK013111	1.416	1.127	−1.043
Dppa2	1453223_s_at	AK010743	1.415	1.327	1.112
Fbxl20	1456378_s_at	AV120094	1.356	1.110	−1.102
Moap1	1448787_at	BC014715	1.346	1.132	1.076
B3galt3	1418736_at	BC003835	1.343	−1.839	−1.844
Khsrp	1436813_x_at	BB332580	1.336	−1.043	−1.26
BC037674	1434835_at	BM230523	1.309	1.007	1.134
Mrpl15	1430798_x_at	AV306676	1.307	−1.338	−1.473
D030056L22Rik	1423879_at	BC020125	1.304	1.040	−1.018
Ttc13	1437709_x_at	BB492914	1.270	1.017	1.106
Nfkbia	1420088_at	AI462015	1.225	−1.081	−1.229
Hrb	1426923_at	BB130716	1.215	−1.020	1.034

Numbers correspond to fold change values for identified target genes at selected time points (0–15 and 0–21 hours) in TAG1 and J1 cells (0–12h). At time point 0 TAG1 and J1 cells have been pre-treated with SB for 6 hours to turn-off all Smad2/3 signalling. At 15 hour TAG1 cells have been treated only with Dox and at 21 hours cells have been treated sequentially with Dox for 15 hours followed by SB for another 6 hours. J1 parental cells have been treated for 12 hours only with Dox. Sixty-nine probe-sets coding for 60 target genes are ranked based on the fold change of gene expression at 15 hours (h) under Dox induction.

To exclude the possibility that any of the 64 target genes responds to Dox/rtTA and not to P-Smad, we performed microarray analysis on the parental J1 cells (containing rtTA, but not Alk4*) after 6 and 12 hours of induction with Dox (Table 1 and Table S1). Several genes were upregulated by the rtTA (ArrayExpress database); however, from the list of 64 genes only four showed a significant increase in J1 cells as high as in the TAG1 cells, suggesting that these four genes respond to rtTA rather than to Alk4*-Smad2/3 activation and were removed from the list of Smad2/3 targets. Two genes *GalNAcS-6ST* and *Fgf15* respond to rtTA (Table 1: up to 7.34- and 3.16-fold respectively), but they were upregulated to a greater level in the Dox treated TAG1 cells (Table 1: up to 36.32- and 15.146-fold respectively) and therefore, were considered as Smad2/3 targets.

Among the remaining 60 genes, four (*Pitx2, Nodal, Lefty1* and *Lefty2*) are known direct targets of Nodal during early embryogenesis. *Smad7* and *SnoN* have not been shown to be downstream P-Smad2/3 during early development; however, tissue culture assays indicated that they are direct targets [38], [39]. We therefore identified 54 new P-Smad2/3 candidate direct target genes in ES cells. Several other known Smad2/3 target genes were not found in our screen, because they are either repressed in ES cells or they require additional partner factors, which are not present in ES cells. Alternatively, they do not meet our stringent criteria. As Alk4 and Alk4* may also activate other pathways [40], we cannot exclude the possibility that some of the transcriptional responses may be due to activation of Smad independent signalling. However, as in this study we correlate the level of target gene expression with that of P-Smad2/3 and not Alk4*, we refer to these genes as Smad2/3 targets rather than Alk4* and Smad2/3.

**Figure 2:**
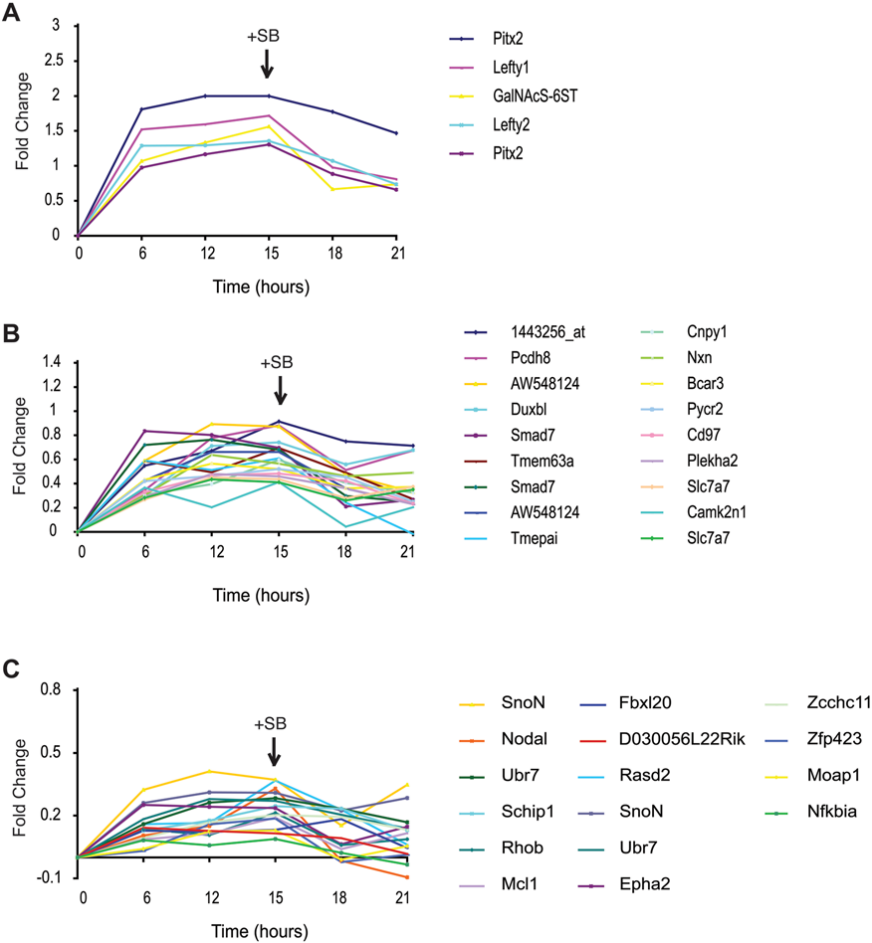
Expression profile of genes responding to the manipulation of Alk4*-Smad2/3 activation in ES cells. Microarray expression analysis of TAG1 ES cells treated sequentially with Dox (activation; 6–15 hours) and SB (inhibition; 18–21 hours). The behaviour of 33 Smad2/3 upregulated genes at different time points (x-axis) during the treatment is represented with trend lines. All samples were normalised to control time points (0) and the relative level of gene expression is presented as the log values of fold change (the y-axis). For all values see Table S1. (A) Trend lines showing the expression profile for genes classified as high response targets (≥10-fold upregulation in gene expression at time point 15 hours). (B) Trend lines showing the expression profile of a sub-set of medium response genes, which increases by ≥2.5- to <10-fold. (C) Trend lines for a subset of low sensitivity response genes, which are upregulated by ≥1.2- to <2.5-fold.

We analysed the expression profile for the 60-upregulated target genes throughout the course of the activation and inhibition of Alk4*-Smad2/3. The microarray data for each gene was plotted as trend-lines showing the fold-change in log scale compared to the time point 0 (SB pre-treated cells), when there is no detectable P-Smad2/3 (Figure 2). We classified the upregulated genes in three main groups based on their fold-change at time point 15 (highest value): “high response”, “medium response” and “low response” (Table 1 and Figure 2). Notably, this classification inversely reflects the basal level of expression of the target genes at time point 0, when Smad2/3 activation has been inhibited with SB for 6 hours. Therefore, genes with very low basal levels of expression (Table 2) show the highest fold change in response to Alk4*-Smad2/3 activation, suggesting that in ES cells the expression of these genes depends solely on P-Smad2/3. In this group we found 5 genes (6 probes) that show an upregulation higher than 10-fold (Figure 2A). For example *Pitx2* is upregulated as much as 100-fold and *Lefty1* by 52-fold. In this group we also found *GalNAcS-6ST* (4631426J05Rik), a sulfotransferase, which has not been shown to be a P-Smad2/3 target. However, its pattern of expression during early development (gastrulation) is consistent with being a Nodal regulated gene [41].

**Table 2:** Basal levels of expression of Smad2/3 target genes in ES cells

Gene	CHX	Dox/SB15	Dox/SB12
Duxbl	0.165	0.116	0.073
1443256_at	0.397	0.172	0.260
Cnpy1	0.206	0.108	0.135
Camk2n1	0.383	0.239	0.170
GalNAcS-6ST	0.024	0.026	0.058
Ubr7	3.045	3.846	3.620
Ubr7	1.750	2.306	2.020
5730419I09Rik	1.045	0.712	0.609
Aasdhppt	2.216	2.144	1.662
Abcg2	1.924	1.677	1.008
Gpr107	1.820	0.787	0.093
Atrx	0.964	0.693	0.335
AW548124	0.468	0.229	0.147
AW548124	0.089	0.078	0.057
B3galnt1	0.421	0.411	0.220
Bbc3	1.316	0.857	0.273
BC037674	5.230	4.315	2.803
Bcar3	1.028	0.597	0.432
Bhlhb8	0.367	0.250	0.059
Ccnd2	1.599	0.699	0.551
Ccnd2	1.025	0.471	0.208
Cd97	1.019	0.426	0.656
Cripto	3.506	4.914	3.064
D030056L22Rik	2.981	2.192	1.021
D6Wsu176e	2.482	3.764	3.057
Dppa2	6.387	6.426	4.827
Dusp9	1.995	0.914	0.893
Dusp9	1.978	0.887	0.846
Eif3s6ip	1.172	0.854	0.523
Epha2	2.271	2.591	1.436
Fbxl20	3.094	1.753	0.400
Fgf15	0.596	0.117	0.055
Hrb	2.794	2.951	1.841
Khsrp	1.972	1.233	1.097
Lefty1	0.366	0.531	0.054
Lefty2	0.073	0.160	0.057
Lgr4	2.608	1.822	1.890
Mcl1	5.911	8.708	6.379
Moap1	2.283	3.018	2.214
Mrpl15	1.469	1.519	0.691
Nfkbia	5.817	4.291	2.193
Nodal	1.448	1.196	1.502
Notch3	1.581	0.990	0.297
Nphs1	1.046	1.521	1.363
Nxn	1.788	0.559	0.675
Pcdh8	0.247	0.090	0.056
Pea15	2.070	0.957	0.666
Pitx2	0.031	0.025	0.061
Pitx2	0.277	0.083	0.096
Plekha2	0.503	0.447	0.272
Ppp1r2	2.998	2.632	2.404
Pycr2	1.927	2.978	2.758
Rasd2	0.418	0.376	0.125
Rhob	1.645	1.230	0.634
Schip1	2.863	3.048	2.337
Ski	1.772	1.506	0.928
Slc7a7	2.619	1.881	1.236
Slc7a7	4.194	3.241	2.620
Smad7	0.904	0.523	0.359
Smad7	0.330	0.191	0.147
SnoN	4.403	3.634	3.292
SnoN	0.505	0.403	0.176
Sntb2	1.049	0.668	0.396
Tmem63a	0.187	0.195	0.134
Tmepai	0.406	0.211	0.191
Tmepai	0.690	0.242	0.189
Ttc13	2.465	1.757	1.376
Zcchc11	3.293	1.876	1.015
Zfp423	1.953	1.948	1.383

Sixty-nine probe sets coding for 60 target genes are listed in alphabetical order. Numbers correspond to intensity values at time point 0 of each experiment Dox/SB15 (Table 1 and Table S2), Dox/SB15 (Table S2) and CHX (Table 3). At time point 0 TAG1 cells have been pre-treated with SB for 6 hours to turn-off all Smad2/3 signalling.

The medium response group consists of 16 genes (19 probes) that show a moderate upregulation (≥2.5- and <10-fold; Figure 2B). The 39 remaining genes (45 probes) are weakly upregulated (≥1.2- to <2.5-fold) and form part of the low response group (Figure 2C). Notably, this last group includes the known direct target *Nodal* (fold-change 2.1) and *SnoN* (fold change 2.3) indicating that the rest of the genes of this group have good probability to be direct targets. The low fold-change implies that they have high basal levels of expression at time point 0 and therefore, are also regulated independently of Alk4*-Smad2/3. Different probe-sets coding for the same gene were consistently found within the same group, except for the gene *Tmepai*, which has 3 probe in two different groups and belongs to both categories. In addition, the majority of the 60 genes are reproducibly grouped into the same category in the two different Dox/SB experiments (Table S1 and S2). Only 9 genes switched category between experiments (highlighted genes in Table S2).

Using different methods such as semi-quantitative and quantitative-PCR we confirmed the expression pattern of a subset of genes included in our list (Figure S2). We concluded that the identified 54 novel genes are candidates for being direct, as they reproducibly and readily follow the activation patterns of Smad2/3 over time.

### Direct P-Smad2/3 transcriptional responses occur by de-repression of Alk4* in the absence of protein synthesis

**Figure 3:**
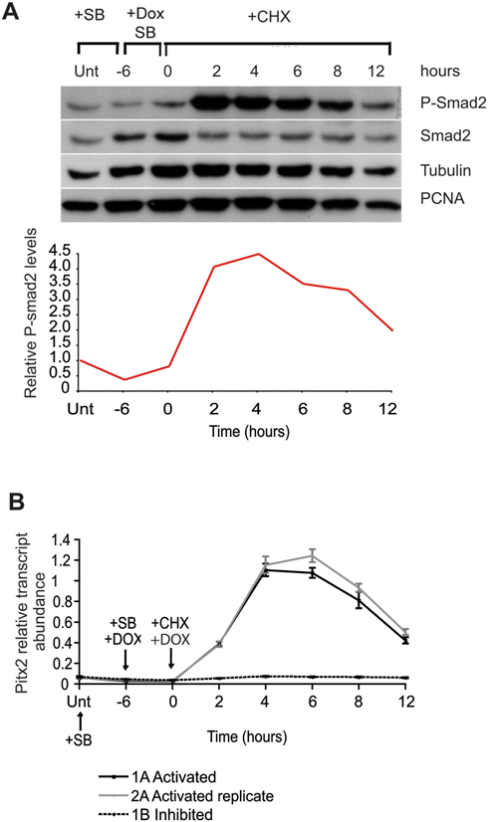
Expression profile of *Pitx2* downstream a Smad2/3 activation time-course in the absence of protein synthesis. (A) Western blots of extracts derived from TAG1 ES cells untreated (unt); pre-treated with SB inhibitor for 6 hours (−6); and under SB for 12 hours but given Dox for the last 6 hours to also induced (Dox) and synthesised Alk4* receptor protein (0). The rest of the samples are extracts from cell treated as at time “0” but subsequently de-repressed for different time periods (2), (4), (6), (8) and (12) hours by removal of SB in the presence of CHX protein synthesis inhibitor. Protein levels were analysed with antibodies against P-Smad2, Smad2 and Tubulin, PCNA controls. The curve chart shows densitometry measurements of the P-Smad2 bands normalised against Tubulin (control). All values are expressed relative to the untreated control (Unt.) represented as 1.0 (B) Real-time PCR data showing changes in the expression levels of endogenous *Pitx2* during the manipulation of Smad2/3 phosphorylation in the absence of protein synthesis (curve 1A and 2A derives from biological replicate experiments under CHX with Smad2/3 activatio, while 1B derives from cells under CHX and SB inhibited in all time points). Relative *Pitx2* transcript abundance is expressed as the average of 4 PCR reactions (n = 4) normalised to the average expression of housekeeping controls *Gapdh*, *Ube*, *Ywhaz* and *B2m* with standard error of the mean of PCR reactions.

To examine how direct the response of the identified genes is, and to ensure that they are immediate targets of Alk4*-Smad2/3 activation we performed the activation of Smad2/3 in the absence of protein synthesis. This was achieved using cycloheximide (CHX) a protein translation-elongation inhibitor. We pre-treated the TAG1 cells with SB inhibitor for 6 hours (time point −6) and subsequently for another 6 hours (time point 0) with Dox in the presence of SB. During this treatment signalling was inhibited for 12 hours while Alk4* protein was produced in the last 6 hours of this period in TAG1 cells with minimal activation of Smad2/3. Subsequently, we removed the SB inhibitor and added CHX to allow phosphorylation of Smad2/3 by the accumulated Alk4* preventing any novel translation from the activated downstream Smad2/3 target genes. Under these conditions we were able to follow over a period of 12 hours the resulting primary transcriptional responses downstream Smad2/3 activation (Figure 3).

Western blot analysis of P-Smad2 levels at different time points during this experiment, showed minimal activation of Smad2 by Dox treatment in the presence of SB inhibitor (Figure 3A, −6 to 0 hours), whereas P-Smad2 reached maximum levels only 2 hours after removal of SB in the presence of CHX (Figure 3A). P-Smad2 levels stayed at maximum for 4 hours and were gradually depleted (Figure 3A) most likely due to decay of the ALK4*. protein pool. Phosphorylation of Smad2 was not observed in a control experiment where SB inhibitor was maintained throughout the experiment in the presence of CHX (data not shown). These results indicate that Smad2/3 was activated efficiently and highly in the absence of protein synthesis.

We followed the transcriptional responses downstream Smad2/3 activation in the absence of protein synthesis by analysing the expression of the direct target gene *Pitx2*, by quantitative PCR (Figure 3B). We found that in two different biological replicate experiments *Pitx2* was activated as early as 2 hours after Alk4* de-repression (removal of SB). Its expression continued to increase at 4 hours (Figure 3B, lines 1A and 2A) and gradually declined after that following the decrease in P-Smad2 levels. *Pitx2* was not activated in the cells where SB inhibitor was maintained throughout the experiment in the presence of CHX, indicating that it responded to Smad2/3 activation and not to CHX (Figure 3B, line 1B). Therefore, this system can be used to identify the primary and immediate early transcriptional responses downstream Smad2/3 activation in ES cells and to assess the sensitivity of their response.

### Thirty-three out of the sixty target genes responded to Smad2/3 activation in the absence of protein synthesis

To identify which of the 60 target genes were activated by Smad2/3 without the requirement of intermediate proteins and in the absence of feedback factors, we performed microarray analysis of mRNA at different time points under the conditions described above (Alk4* de-repression upon removal of SB in the presence of CHX). We found that 33 out of 60 genes were upregulated by at least 1.2-fold (P>0.01) at 2 or 4 hours after Alk4* de-repression (Table 3), when P-Smad2 levels were highest and saturated (Figure 3A). We therefore, considered these genes to be activated directly downstream P-Smad2/3.

**Table 3:** Behaviour and classification of 34 target genes in the absence of protein synthesis

Direct Targets with ≥10.0-Fold Increase in absence of protein synthesis at 0h–4h					
Gene	0h–2h	0h–4h	0h–6h	0h–8h	0h–12h
Pitx2	24.973	57.631	36.798	37.593	12.979
GalNAcS-6ST	9.328	47.469	9.708	7.867	2.411
Lefty1	22.993	36.326	28.388	17.475	3.883
**Direct Targets with 2.5 to 10.0-Fold Increase in absence of protein synthesis at 0h v 4h**					
**Gene**	**0h–2h**	**0h–4h**	**0h–6h**	**0h–8h**	**0h–12h**
Lefty2	1.943	5.779	4.207	4.013	2.021
Smad7	3.697	5.668	3.782	2.698	1.928
Camk2n1	3.843	4.813	2.546	1.961	1.164
Duxbl	2.32	4.743	5.974	8.471	7.473
Tmepai	2.209	3.926	2.44	1.826	−1.244
Tmepai	2.193	3.735	2.665	2.35	1.184
Smad7	3.095	3.436	2.31	1.887	1.606
Rhob	2.517	3.375	3.537	3.155	2.3
Tmem63a	1.256	3.371	3.957	5.154	4.546
Pitx2	1.68	3.079	2.349	1.989	1.401
AW548124	1.091	2.735	1.33	1.216	1.856
**Direct Targets with 1.2 to 2.5-Fold Increase in absence of protein synthesis at 0h v 4h**					
**Gene**	**0h–2h**	**0h–4h**	**0h–6h**	**0h–8h**	**0h–12h**
SnoN	1.527	2.435	1.499	1.364	1.063
Nodal	2.187	2.358	2.076	1.493	−1.768
1443256_at	1.851	2.337	2.073	1.583	1.267
Rasd2	1.137	2.144	2.523	2.527	2.172
SnoN	1.406	2.063	2.108	1.947	1.376
Cd97	1.391	2.036	2.587	2.696	2.811
Epha2	2.161	1.981	1.099	−1.454	−2.433
Mcl1	1.564	1.966	1.943	1.768	1.631
Pycr2	1.391	1.853	1.963	2.44	2.293
Nfkbia	1.565	1.654	2.14	1.842	1.904
Moap1	1.482	1.634	1.667	1.199	1.364
Bcar3	1.358	1.609	1.858	1.671	1.262
Pcdh8	1.256	1.491	1.371	1.317	1.399
Ubr7	1.224	1.485	1.714	1.744	1.496
D030056L22Rik	1.25	1.414	1.115	−1.158	−1.198
Cnpy1	1.175	1.405	−2.408	−2.164	−2.409
Plekha2	1.08	1.346	1.591	1.803	1.846
Zcchc11	1.089	1.287	1.088	−1.071	1.019
Schip1	1.138	1.286	1.306	1.048	−1.055
Slc7a7	1.161	1.265	1.267	1.034	1.063
Zfp423	1.033	1.254	1.366	1.221	1.007
Ubr7	1.165	1.236	1.417	1.464	1.453
AW548124	1.04	1.232	1.05	1.149	1.416
Fbxl20	1.023	1.23	1.439	1.464	2.185
Nxn	1.044	1.203	1.405	1.558	1.136

Fold-change of expression for 38 probe sets coding for 33 target genes at indicated time points after removal of SB and Smad2/3 activation in the absence of protein synthesis compared to the time point 0 hours. At time point 0 the TAG1 cells have been pre-treated with SB for 6 hours and with SB + Dox for an additional 6 hours. Genes are classified in descending order with the genes showing the strongest upregulation 4 hours after activation of Smad2/3.

**Figure 4:**
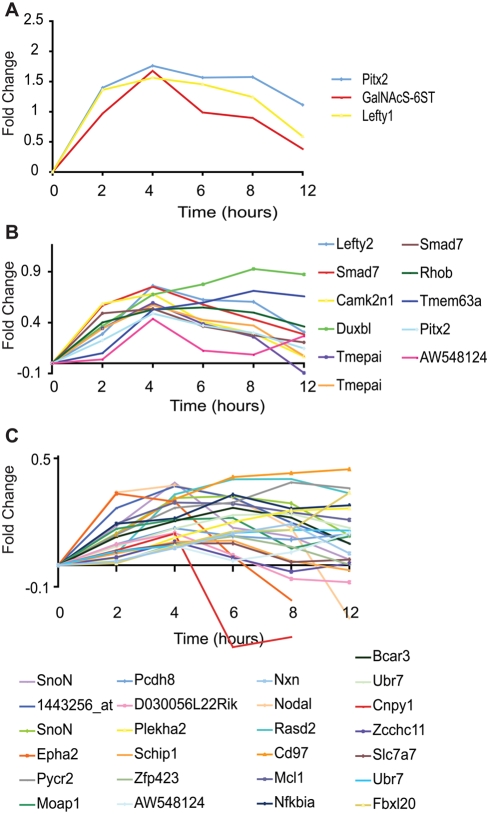
Expression profile of target-genes downstream a Smad2/3 activation time-course in the absence of protein synthesis. Microarray data from TAG1 ES cells under Smad2/3 activation in the absence protein synthesis, as described in Figure 6. The expression of individual genes was analysed and plotted as trends over time. Relative level of gene expression is represented as the log values of fold change (the y-axis) against time (x-axis). All samples were normalised to the time point 0. Genes are classified in 3 groups according to the level of response at time point 4 hours compared to 0. (A) High responding genes, showing ≥10.0-fold increase. (B) Medium responding genes, showing ≥2.5- to <10.0-fold increase. (C) Last group comprising the low responding genes (≥1.2 to >2.5- fold increase). For all values see Table 3.

This new list of genes includes all the known Nodal/Smad2/3 direct targets present in the list of 60 genes (i.e. *Pitx2, Lefty1, Lefty2, Smad7, SnoN and Nodal*), confirming that the conditions and criteria of the experiment are appropriate for the identification of direct target genes. We classified these 33 target genes into the same 3 categories of responses (high, Figure 4A; medium, Figure 4B; and low, Figure 4C), as in the previous Dox/SB experiments, based on the fold of induction at 4 hours (Table 3). In the absence of protein synthesis *Pitx2* (57-fold), *GalNAcS-6ST* (47-fold) and *Lefty1* (36-fold) also reached high levels of expression and were classified, as before, in the high response group (Figure 4A). In general, the fold of induction was weaker in the presence of CHX, compared to the induction in the presence of Dox and protein synthesis, suggesting that peak levels were not achieved. This could be because co-factors downstream Smad2/3 are required for their activation or for mRNA stability. However, two genes, *Rhob* and *Camk2n1* had a higher fold of induction in the absence of protein synthesis (compare Table 1 and Table 2). This could be because in the presence of protein synthesis these genes are under a moderate repression or because their RNA is unstable by a gene specific negative feedback. As SB pre-treatment of TAG1 cells for 12 hours may not have eliminated completely Smad2/3 phosphorylation, some residual feedback factor may have been produced and remained stable during the CHX treatment. However, such leaky expression of secondary factors is expected to be low and die off quickly in the absence of protein synthesis. Such factor could produce mostly weak expression of secondary target genes at levels, which are below the cut-off point (<1.2 fold) after 4 hours of de-repression (P-Smad2/3 activation). In conclusion, our study revealed that among the 60 identified Smad2/3 target genes, 33 (27 novel) are most likely activated directly, whereas the rest (Table S3) do not show significant upregulation suggesting that they require intermediate proteins and/or partners factors for their activation.

### FoxH1/Smad2 elements and Smad binding sites are present in the identified target genes

The Smad2/FoxH1 signalling pathway acts through FoxH1 binding at defined elements [32], [33]. The known Smad2/3 target genes *Nodal, Lefty2 and Pitx2* contain pairs of FoxH1 binding sites separated by 30–200 bps [33], [42], [13], [43] these were termed asymmetric elements (ASEs) as they drive Nodal-dependent asymmetric gene expression. To investigate whether our Smad2/FoxH1 candidate direct target genes contained ASE-like sequences, we undertook a bioinformatics analysis. Mouse genomic sequence, encompassing loci plus 10kb each of upstream and downstream sequence, was screened for pairs of FoxH1 binding sites within 30–200 bps of each other; all possible sequence orientations were tested (as described in the Materials and Methods). According to this definition, 19 of 39 loci examined contained an ASE (Table S4), suggesting that these genes respond to Smad2/FoxH1 through an ASE and they are direct targets of Nodal signalling. There was, however, no correlation between the number of putative ASEs and the levels of expression.

Multispecies sequence comparison has previously demonstrated conservation of functionally important transcription factor binding sites [44], [45]. If the putative ASEs that we have identified are significant *in vivo*, similar sequence conservation would be expected. Therefore, MultiPipmaker analysis was used to compare the putative mouse ASE sequences with the corresponding sequences from human, chimp, dog and rat. Pairwise alignments of sequence from these species with the mouse sequence were computed and the resulting alignments were summarised as “percent identity plot” or “Pipplot” (Figure S3). Three different levels of putative ASE conservation were observed: high cross species conservation was seen for the known Smad2/FoxH1 targets (*Nodal*, *Pitx2*, *Lefty1* and *Lefty2*) as well for one putative ASE in *Zfp423* (Figure S4). Interestingly, the predicted ASEs of the majority of potential novel Smad2/FoxH1 targets were conserved specifically in rodents suggesting that in other species these sites have changed position or got lost. A small number of putative ASEs showed no cross species conservation at all (Figure S4).

Identified as components of the TGFβ inducible elements in the PAI-1 locus, CAGA boxes can bind both Smad3 and 4 [46]. The presence of CAGA boxes was screened for in 33 loci that were directly regulated by Smad2/3 in the absence of protein synthesis, and an additional 6 loci that were only upregulated in the presences of protein synthesis (Table S4). As expected for a short single sequence, many CAGA boxes were predicted in most of the 39 genes, irrespective of the presence of ASEs. Notably, no CAGA boxes were predicted at *Pitx2*, a locus with previously characterised ASE elements. Similarly, no close physical proximity was detected between ASE elements and CAGA boxes in those genes where putative ASE elements were predicted. The above data support the hypothesis that most of the identified target genes are regulated directly by Smad2/3FoxH1 and therefore are direct Nodal targets.

### The expression of direct target genes depends on Nodal signalling in the early embryo

**Figure 5:**
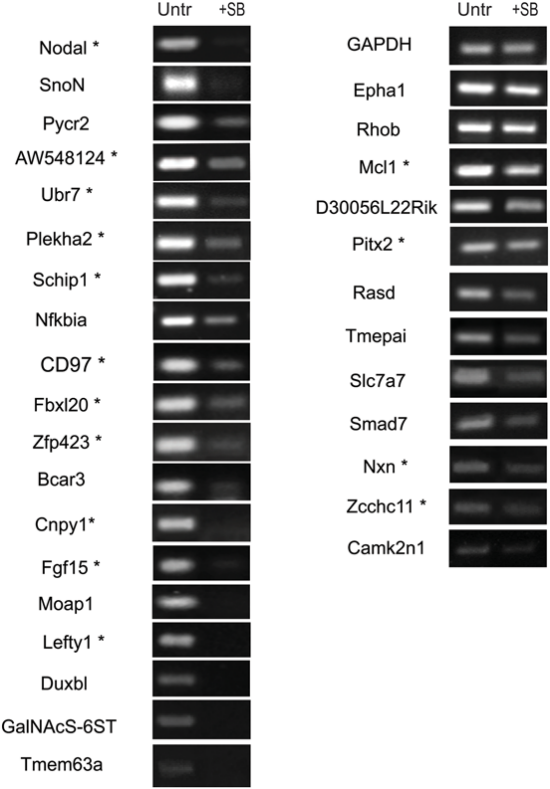
The identified direct target genes are expressed in the mouse embryo under Nodal/Smad2 signalling activation. RT-PCR on identified direct target genes from E5 embryos cultured with inhibitor (SB) or without (Untr) for 18 hours. All the tested genes are expressed in uninhibited embryos (Untr). Like *Nodal* (positive control) the majority of identified target genes are downregulated under SB treatment. No effect on *GAPDH* (negative control) expression shows that SB does not generally inhibit gene transcription in cultured embryos. Genes marked with (*) contain predicted Foxh1 binding elements (ASE).

The majority of the novel target genes identified in our system, have not been studied at early developmental stages. Nodal/Smad2/3 signalling is essential for gastrulation and patterning of the anterior posterior axis of the vertebrate embryo. To investigate the extent to which the identified target genes are relevant to Nodal signalling in the embryo, we examined their expression in early mouse embryos (embryonic day 5, E5, and 6, E6) when Nodal signalling is active. The expression of the target genes was examined using RT-PCR. In addition, we tested whether the expression of these genes is dependent on Nodal signalling, by culturing E5 embryos in defined medium with or without SB inhibitor for 18 hours prior to RT-PCR analysis (Figure 5).

We observed that all novel target genes are expressed at this stage and that inhibition of the Nodal pathway in the embryos (SB treatment) results in significant downregulation of 19 genes including *Nodal*. The remaining genes were weakly- or un-affected by the inhibition of the pathway in embryos. This may be due to the sensitivity of the RT-PCR or the presence of other factors that maintain the expression of these genes in the embryo via a Nodal independent mechanism. Another possibility is that very low levels of signalling might be still active in SB-treated embryos, sufficient to maintain the expression of these specific genes. Interestingly, the majority of these genes contain FoxH1/Smad2 binding sites (indicated by an asterisk in Figure 5) adding supporting evidence that they are direct Nodal targets. Collectively, the above data show that the ES cell system resembles the transcriptional status of early embryonic development and suggests that the novel target genes identified here are regulated by Nodal signalling in the embryo.

### The TAG1 database can be used to investigate additional expression patterns downstream Smad2/3 activation

**Figure 6:**
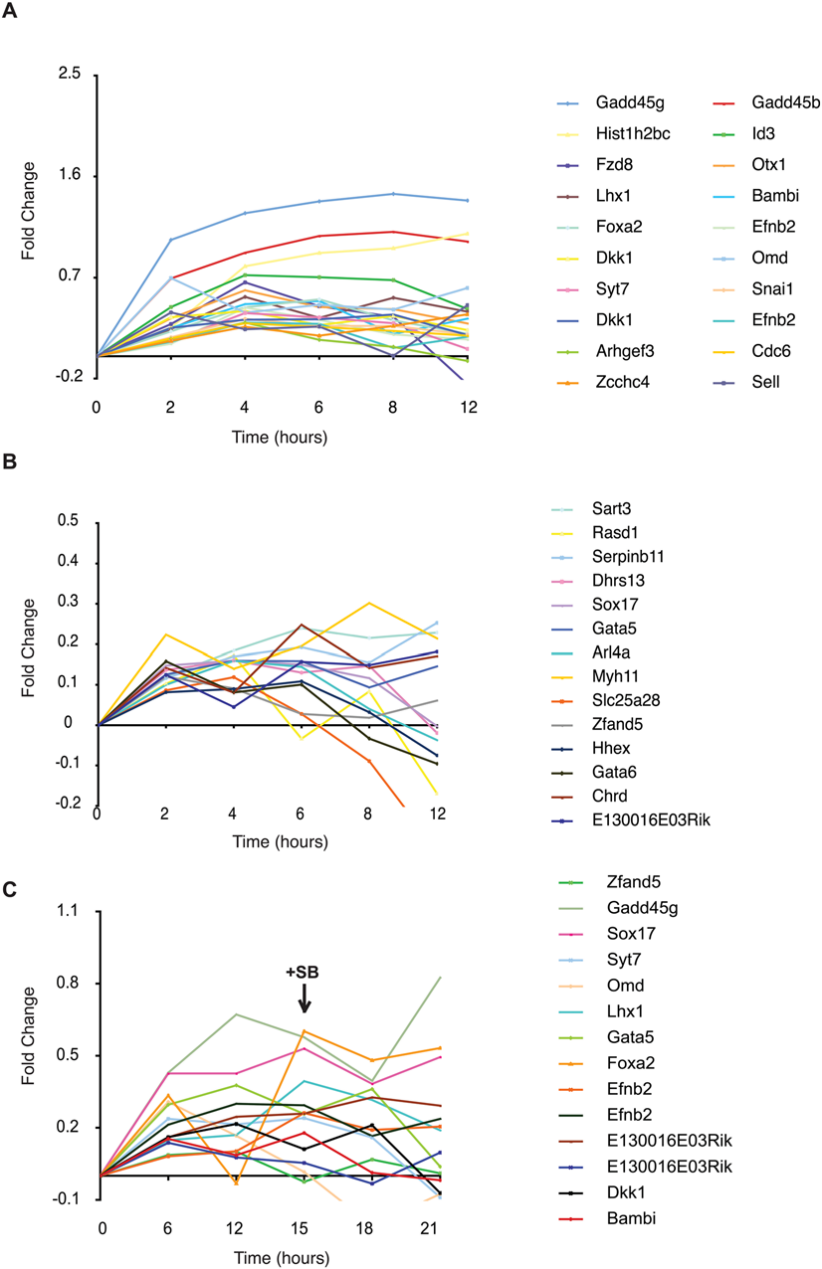
Expression profile of known Nodal-regulated genes in the TAG1 Smad2/3 time-course databases. Gene expression patterns of known Nodal-regulated genes were analysed and plotted as trends to show the changes in the expression during the modulation of Smad2/3 in the absence (A and B) or presence (C) of protein synthesis. Relative level of gene expression is represented as fold-change (y-axis) in log scale against time (x-axis). All samples were normalised to the 0 time point. Genes with at least one set of probes upregulated (≥2-fold) 2 hours after Smad2/3 activation are shown in (A and B) and genes with one set of probes upregulated (≥1.2-fold) 6 hours after Smad2/3 activation in the Dox/SB15 experiment are shown in (C). The values of fold-change for the genes in these graphs are shown in Tables S5 and S6.

Recent genome-wide screens in vertebrates and tissues culture assays have increased the list of genes regulated by Nodal-related ligands during embryogenesis and other cellular contexts [47], [7]–[9], [6]. However, it is not known whether these genes respond directly to Nodal signalling over time. We took advantage of our TAG1 databases to examine the expression profile of at least 150 genes that have been previously identified to be downstream of Nodal. We initially examined the expression profile of these genes in the CHX database, which contains genes that are activated by Smad2/3 in the absence of protein synthesis. We released the p-value constrain from all time points and selected the genes that show greater than 1.2 fold (≥1.2) upregulation in the first 2 hours, and that maintain or increase further their expression levels in 4 hours under activation of Smad2/3 in the presence of CHX. For the genes that are represented by more than one probe on the microarray chip, we selected those that have at least one probe following the above criteria. We found that 32 out 150 genes fulfilled the above criteria (Figure 6A and 6B; Table S5). We then examined the expression profile of these 32 genes in the presence of protein synthesis in the two Dox/SB experiments. We selected genes with upregulation ≥1.2 fold in the first time point, 6 hours with Dox induction (Table S6). Twelve genes out of 32 were found to be upregulated in 6 hours in the Dox/SB15 experiment and 17 in the Dox/SB12 experiment (Table S6). Only 7 genes (*Bambi*, *Dkk1*, *Gadd45g*, *Omd*, *Sox17*, *Syt7* and *Zfand5*) were in common between these databases. Notably, Bambi, a BMP/Activin membrane bound receptor inhibitor [48], and Syt7 (synaptotagmin VII), a calcium sensor protein that regulates exocytosis [49], follow faithfully the activation pattern of Smad2/3 in all time points in both Dox/SB experiments (Figure 6C and Table S6) and under CHX (Figure 6A and 6B) suggesting that they are direct targets.

The rest of the 32 known Nodal regulated genes, include factors with roles during gastrulation such as Chrd, Dkk1, Foxa2, Gata5, Gata6, Hex, Id3, Lhx1, Otx1, Snail1, Sox17 etc [27], [50], [51]. These do not follow the pattern of Smad2/3 activation in ES cells beyond the first 6 hours, in the presence of protein synthesis. However, they all respond to Smad2/3 activation in the absence of protein synthesis (Figure 6A, 6B and Table S5). These genes are normally activated and expressed at later stages in development and not in naïve pluripotent cell environment. Therefore, their expression may require co-factors not present in ES cells. Alternatively, in the presence of protein synthesis, repressors that are activated downstream of Smad2/3 in ES cells could be responsible for suppressing the premature activation of gastrulation specific genes.

In this study we used criteria to identify genes that follow faithfully the activation of Smad2/3 over time, and showed that our database can be used with other criteria to identify target genes that respond differently. For example the TAG1 database includes target genes that are downregulated by Alk4*-Smad2/3 activation in the presence or absence of protein synthesis, etc. (not shown). The above analysis is a proof of principal that our database is a useful resource to screen for gene regulation downstream Nodal signalling in ES cells.

## Discussion

The transcription effectors Smad2/3 regulate several hundreds of genes downstream of TGFβ ligands, including morphogens such as Nodal and Activin with essential roles in vertebrate embryonic development. The concentration of morphogen and the exposure of the responding cells to signalling are critical parameters for specifying cell fate [52], [53]. How the cells convert the concentration of ligand to specific transcriptional responses is unclear. It is believed that the TGFβ morphogen levels are reflected within a cell by the concentration of activated Smad2/3 effectors, which are then responsible for the downstream transcriptional responses. However, feedback regulatory mechanisms (positive and negative) are activated directly by P-Smads over time and alter not only the levels of P-Smads, but also their transcriptional activity. Correlation of effector levels with expression patterns of target genes over time had not been addressed in developmental context.

To obtain insights into this question we generated an inducible system in ES cells to manipulate the levels of Smad2/3 activation intracellularly, bypassing all extracellular feedback. We examined the transcriptional responses at a genome-wide scale at different time points over the course of 30 hours during which activation of Smad2/3 is followed by repression of signalling. The experiment was repeated twice, and only consistent patterns of expression were studied further. Additionally, to address the response of direct target genes in the absence of protein-based feedback mechanism, we performed the activation of Smad2/3 in the absence of protein synthesis and analysed the transcription patterns at different time points.

The most important observations of this analysis (summarized in Table 4) include: (1) the identification of a group of novel target genes with patterns of expression that follow faithfully and reproducibly the activation/repression profile of Smad2/3 in the presence or absence of protein synthesis in ES cells; (2) the finding that among these genes the most readily responsive were main feedback factors; (3) the observation that the majority of target genes follow Smad2/3 activation with similar degree of sensitivity in the presence or absence of protein synthesis (see classification in all three experiments), indicating that intracellular feedback mechanisms acting at the level of P-Smad2/3 activity are ineffective in ES cells; (4) that the identified direct target genes are expressed in the early mouse embryo, under Nodal/Smad2/3 signalling, confirming that the ES cell system is relevant to development; and (5) that most of the direct target genes contain conserved FoxH1/Smad2 (ASE) binding elements supporting that they are direct targets. Furthermore, we were able to examine the expression patterns of known Nodal regulated genes in the database of our experimental system and identify the ones that are upregulated in the absence of protein synthesis. This illustrates how our database can be used for the study of thousands of gene-expression patterns downstream Nodal-Smad2/3 activation time course in ES cells.

**Table 4:** Summary of results for 60 Nodal-Smad2/3 target genes.

Gene	Dox/SB15	CHX	ASE	Expression in E6 embryo	Expression in E6 embryo +SB	Status
1443256_at	M	L	1	N/A	N/A	Novel/Direct
5730419I09Rik	L	-	N/A	N/A	N/A	Novel
Aasdhppt	L	-	N/A	N/A	N/A	Novel
Abcg2	L	-	N/A	N/A	N/A	Novel
Atrx	L	-	N/A	N/A	N/A	Novel
AW548124	M	M	2	++	+/−	Novel/Direct
B3galt3	L	-	N/A	N/A	N/A	Novel
Bbc3	L	-	0	N/A	N/A	Novel
BC037674	L	-	N/A	N/A	N/A	Novel
Bcar3	M	L	0	++	--	Novel/Direct
Bhlhb8	L	-	N/A	N/A	N/A	Novel
Camk2n1	M	M	0	+	+/−	Novel/Direct
Ccnd2	L	-	N/A	N/A	N/A	Novel
Cd97	M	L	2	++	+/−	Novel/Direct
Cnpy1	M	L	1	++	--	Novel/Direct
Cripto	L	-	N/A	N/A	N/A	K
D030056L22Rik	L	L	0	++	+	Novel/Direct
D6Wsu176e	L	-	N/A	N/A	N/A	Novel
Dppa2	L	-	N/A	N/A	N/A	Novel
Dusp9	L	-	N/A	N/A	N/A	Novel
Duxbl	M	M	No	+	--	Novel/Direct
Eif3s6ip	L	-	N/A	N/A	N/A	Novel
Epha2	L	L	0	++	+	Novel/Direct
Fbxl20	L	L	4	++	+/−	Novel/Direct
Fgf15	H	-	1	++	--	Novel
GalNAcS-6ST	H	H	0	+	--	Novel/Direct
Gpr107	L	-	N/A	N/A	N/A	Novel
Hrb	L	-	1	N/A	N/A	Novel
Khsrp	L	-	N/A	N/A	N/A	Novel
Lefty1	H	H	1	++	--	K
Lefty2	H	M	1	N/A	N/A	K
Lgr4	M	-	N/A	N/A	N/A	K
Mcl1	L	L	1	++	+	Novel/Direct
Moap1	L	L	0	++	--	Novel/Direct
Mrpl15	L	-	N/A	N/A	N/A	Novel
Nfkbia	L	L	0	++	+/−	Novel/Direct
Nodal	L	L	1	++	--	K
Notch3	L	-	N/A	N/A	N/A	Novel
Nphs1	L	-	1	N/A	N/A	Novel
Nxn	M	L	4	+	+/−	Novel/Direct
Pcdh8	M	L	0	N/A	N/A	Novel//Direct
Pea15	L	-	N/A	N/A	N/A	Novel
Pitx2	H	H/M	3	++	+	K
Plekha2	M	L	1	++	+/−	Novel/Direct
Ppp1r2	L	-	N/A	N/A	N/A	Novel
Pycr2	M	L	0	++	+/−	Novel/Direct
Rasd2	L	L	0	++	+	Novel/Direct
Rhob	L	M	0	++	+	Novel/Direct
Schip1	L	L	N/A	++	+/−	Novel/Direct
Ski	L	-	0	N/A	N/A	K
Slc7a7	M	L	N/A	++	+	Novel/Direct
Smad7	M	M	N/A	+	+/−	K
SnoN	L	L	N/A	++	--	K
Sntb2	L	-	N/A	N/A	N/A	Novel
Tmem63a	M	M	0	+	--	Novel/Direct
Tmepai	L/M	M	0	++	+	Novel/Direct
Ttc13	L	-	N/A	N/A	N/A	Novel
Novelbr7	L	L	1	++	+/−	Novel/Direct
Zcchc11	L	L	3	+	+/−	Novel/Direct
Zfp423	L	L	5	++	+/−	Novel/Direct

The genes are listed in alphabetical order. The classification high (H), medium, (M) low (L), corresponds to the sensitivity of their response to Alk4^*^-Smad2/3 activation during the time course experiment in the presence (Dox/SB15) or absence (+CHX) of protein synthesis. The presence and number of FoxH1 (ASE) binding sites is indicated. The expression in the embryos with or without SB inhibitor is listed and scored (+, −). N/A, not analysed; Novel, identified target; K, previously known target;

### Efficient manipulation of Smad2/3 activation in ES cells

Smad2/3 activation in ES cells is controlled by the presence of autocrine signalling but also by extracellular feedback mechanisms (i.e. agonists, antagonists, co-receptors etc), which alter the intracellular level of activated Smad2/3 over time. The inducible system we developed in this study is based on the tetracycline inducible Alk4*, which bypasses all extracellular feedback. More importantly, it allowes us to assess transcriptional responses in the absence of protein synthesis and presumably in the absence of all protein based feedback mechanisms. The latter was achieve by Alk4* (Dox) induction in the presence of SB inhibitor and subsequent activation of Smad2/3 phosphorylation by removing SB in the absence of protein synthesis inhibition (CHX). Under these conditions, transcriptional responses were observed as early as 2 hours after Alk4*-Smad2/3 derepression (Figure 3A). The response of the target genes was correlated to different levels of Smad2/3 without protein translation of putative intermediate feedback factors. However, as microarrays is a semi-quantitative method, quantitative PCR is needed to allow accurate correlation in future experiments. Moreover, evaluation of transcription rate and mRNA stability should also be studied, as they can also modulate the sensitivity of the transcriptional responses. Such studies may reveal mechanisms of differential gene expression downstream specific concentrations of P-Smad2/3. Finally, as our ES cell system is relevant to development it could be used to manipulate signalling at different periods during differentiation *in vitro* and lead to the identification of key target genes in different cellular context.

### Identification of novel Smad2/3 primary target genes in ES cells

It has become apparent that the regulation of genes involves combination of transcription factors and enhancer elements, conferring tissue specificity and response to signalling effectors. This means that the target genes downstream signalling are expected to be different and specific to a cell type or tissue. It is therefore important to screen for Nodal/Smad2/3 target genes in ES cells as these represent a developmental relevant context. Here we identified 54 novel gene that follow faithfully and reproducibly the changes of Smad2/3 activation. More than half of these target genes (33 total, 27 novel) were also significantly upregulated after only 2 hours of Smad2/3 activation in the absence of protein synthesis, confirming that they are primary targets and not secondary (which depend on the activation of intermediate transcriptional activators and co-factors). In addition, we showed that these genes are expressed in the mouse embryo at stages where Nodal signalling is active; and are downregulated when Nodal signalling is inhibited (with SB treatment of the embryos), supporting the hypothesis that they are Nodal-Smad2/3 targets relevant to developmental events. Furthermore, the fact that these primary target genes contain conserved FoxH1/Smad2 binding elements (also known as ASE and ARE [32], [33]), strengthens the hypothesis that they are directly regulated by Nodal signalling. However, as Nodal-Alk4* signalling can phosphorylate additional effectors other than Smads [40], we cannot exclude that some of these targets may be Smad independent.

### Duration of Nodal-Smad2/3 signalling and its effect on transcriptional responses

Duration of signalling is an important parameter for the transcriptional responses downstream of Nodal-Smad2/3, as primary early target genes include feedback proteins, which affect the strength of subsequent signalling can reset the pattern of expression over time. The list of genes that are reproducibly activated in the two Dox/SB experiments includes *Nodal* itself and its co-receptor *Cripto* [54] as well as *Nodal* antagonists *Lefty1/2* [55] and *Bambi* (BMP and Activin membrane-bound inhibitor; ([56] and reviewed in [57], [30]). These genes are part of the extracellular feedback mechanism of TGFβ signalling and their role is to change the levels of Smad2/3 activation. In our inducible system, however, extracellular factors are bypassed as activation of Smad2/3 depends on Alk4* induction by Dox. Western blotting confirmed that maximal activation occurs 3–6 hours after Dox treatment (Figure 1C), and is maintained at peak levels over a long period (24 hours Figure S1).

Early-activated genes in our system also include negative intracellular regulators of TGFβ signalling, such as inhibitory Smad7 (I-Smad7) and the co-repressors SnoN and Ski. I-Smad7 has been proposed to interfere with Smad-DNA complex formation [61], which is downstream of the levels of Smad2/3 activation, while SnoN (Ski-like) interacts directly with Smad2/3 and Smad4 and blocks target gene transcription [62], [63]. These negative regulators act on activated Smad2/3 and therefore are expected to reduce target gene transcription with duration of high signaling. However, this was not observed in our system, as the identified target genes are not repressed at 12 or 15 hours under induction. Furthermore, in the absence of protein synthesis, where there are no or little protein dependent feedback mechanisms target genes responded with the same sensitivity as in the presence of protein synthesis (Table 1 and Table 3). Longer periods (>15 hours) of Smad2/3 activation in the presence of protein synthesis is needed to address when the negative regulators repress P-Smad transcriptional activity. Notably, in the experiment where Dox was maintained up to 30 hours in the TAG1 cells, *Nodal* expression (downstream Smad2/3 activation) peaked at 12–20 hours and declined slowly after that (Q-PCR; Figure S1B). As P-Smad2 levels are maintained high throughout this experiment (Figure S1A) the cause of the downregulation at 30 hours is most likely caused by intracellular negative feedback mechanisms, which commence after a 24-hour duration of signalling in ES cells.

The levels of I-Smad7 and co-repressor SnoN are also regulated at the protein level by ubiquitin ligases such as Arkadia, Smurf2 and Anaphase Promoting Complex [64]–[68]. Arkadia is present in ES cells [19] and it controls the levels of Ski/SnoN and I-Smad7 and cancels their negative feedback. We did not find evidence that Arkadia expression is regulated by Smad2/3 (TAG1 database) and therefore, we concluded that this mechanism is present in ES cells and independently of Alk4*-Smad2/3 signalling. Interestingly, we do not find positive regulators, such as partner transcriptional factors FoxH1 and Mixl1, to be activated downstream of Smad2/3 in ES cells. Nevertheless, FoxH1 is already present in ES cells [19] and the majority of the genes on our list contain FoxH1 binding sites. As partner factors determine target gene specificity and are not subject to Smad2/3 regulation, they represent important components of target gene selection in a specific cell context. Therefore, ES cells show bias towards FoxH1 target gene activation and corresponding cell fate [9] suggesting that ES cells are pluripotent but not naïve.

Collectively our analysis suggests that in ES cells and most likely during development, graded levels of activated Smad2/3 effectors are converted proportionately into several target gene expression and that these responses remain sensitive and reversible over a 24 hour period. However, maintenance of signalling over long time leads to the activation of secondary and tertiary transcription factors. These can cause cross-repression or cross-enhancement of primary genes or activate new genes further downstream. Notably, *Pitx2*, an immediate early transcription factor target whose expression is solely dependent on Smad2/3 activation in ES cells, is expected to be involved in major downstream transcriptional effects. Further long-term experiments are required to address when and how the Smad2/3 transcriptional responses become desensitised and fixed leading to a particular cell fate. However, our study is in a population of cells, and the culturing conditions most likely favour maintenance of the ES cells undifferentiated character (self-renewal and proliferation). Single cell analysis and culturing under differentiation condition of the TAG1 induced cells should be more informative on lineage commitment mechanisms downstream Smad2/3 signalling.

Our system can be used in the future for studies of different expression patterns downstream Nodal-Smad2/3 activation (i.e. downregulated genes) and also for studies under different culturing conditions i.e. long exposure to low versus high levels of Smad2/3 activation; or in combination with a different signalling pathway stimulation; etc. Such studies will shed light on the understanding of how time and level of the Nodal/TGFβ effectors select target genes. As Smad2/3 signalling is involved in several functions, from ES cell pluripotency to differentiation towards lineages including endoderm, and in diseases like cancer, our system and results will be useful to a range of scientists addressing diverse subjects.

## Materials and Methods

Αll experiments done on animals were performed under a UK Home Office Animal licence and approved by the Imperial College ethical review committee.

### Cell culture/Derivation of TAG1 cell lines

TAG1 ES cells were generated by co-electroporation of two constructs into feeder free doxycycline inducible J1 ES cells (gift of Anton Wutz, Austria). The constructs were: the *pSLTT-AIG* linearised at the PvuI sites and the *pTriEx-2 hygro* construct (Novagen, UK) linearised at the MluI site and were represented 10∶1 ratio in the total 25 µg of DNA electroporated. The electroporation was performed with 20×10^6^ cells at 0.2kV and 960 µF on the Gene Pulser System (Bio-rad, UK). The J1 cells were selected in ES cell medium: 15% FCS in DMEM (Invitrogen, UK) supplemented with LIF (homemade) (ES cell medium) and selected wtih 110 µg/ml hygromycin (Invitrogen) 48 hours after electroporation. ES cell colonies were picked and screened for inducibility by ES cell medium containing 1 µg/ml of doxycycline for 18 hours. Colonies were detection for GFP fluorescence under UV on a Leitz DMIRB microscope (Leica Microsystems, UK). Clones were picked and expanded. The TAG1 ES cell line was maintained feeder-free in 20% FCS in DMEM supplemented with LIF.

Manipulations of Smad2/3 signalling/activation in TAG1 ES cells were performed under chemically defined conditions using DMEM supplemented with 20% KSR (KSR media). Induction of the TAG1 ES cells was performed using KSR medium supplemented with 1 µg/ml doxycycline (Clontech, UK). Inhibition of the TAG1 ES cells was performed using KSR medium containing 10 µM SB-431542 (Sigma, UK and gift from GSK, UK). As DMSO was used to dissolve doxycycline, the control ES cells were treated with 0.1% DMSO (Sigma, UK). For the identification of direct transcriptional targets, the TAG1 ES cells were cultured in KSR media containing 10 µM SB-431542 and 1.5 µg/ml doxycycline for 6 hours to accumulate Alk4* receptors while inhibiting their activity. This medium was then replaced with fresh containing only 100 µg/ml cycloheximide (Calbiochem, UK). In the uninduced control experiment, the TAG1 ES cells were treated with 100 µg/ml cycloheximide and 10 µM SB-431542.

### Western Blotting

Immunochemistry was carried as described before [19]. Primary antibodies used were: rabbit anti-P-Smad2 (1∶2000) (Calbiochem, UK), rabbit anti-Smad2 (1∶2000) (Zymed Laboratories, USA), rabbit anti-P-Smad3 (1∶1000) (Cell Signaling Technology, USA) or mouse anti-PCNA (1∶5000) (Santa Cruz Biotechnology, USA). Secondary antibodies were: HRP conjugated anti-rabbit (1∶2000) (GE Healthcare, UK) or anti-mouse antibody (1∶5000; Santa Cruz Biotechnology, USA) Quantitation of protein bands were performed on scans of the films and measurements of pixel intensity for each band on Photoshop 7.0 (Adobe Systems Inc., USA).

### FACS sorting

ES cells were harvested using trypsin (Invitrogen), gently dissociated into a single-cell suspension, and resuspended in ice cold PBS at a density of 1×10^5^ cells/ml. FACS analysis was carried out on the FACScan Flow Cytometry System (Becton, Dickinson and Company, USA) using the CellQuest analyser program.

### Microarray analysis

Total RNA was extracted from cells using the RNeasy Mini Kit. Concentration and quality of the RNA was checked on the NanoDrop® ND-1000 Spectrophotometer (NanoDrop Technologies, USA) and the RNA 6000 Nano LabChip® Kit (Agilent Technologies, UK) on the 2100 Bioanalyzer (Agilent Technologies). 10 µg of total RNA for each sample was reverse transcribed using SuperScript™ II Reverse Transcriptase (Invitrogen) following the manufacturer's protocol. The first cDNA strand reaction was used for second cDNA strand synthesis with DNA Ligase, DNA Pol I, dNTPs and RNase H (Invitrogen). The double stranded cDNA was further purified using the GeneChip Sample Cleanup Module (Affymetrix, UK). The double stranded cDNA was then transcribed into biotin labelled cRNA using the Bioarray High Yield RNA Transcript Labelling Kit (Enzo Diagnostics, USA) according to the manufacturer's protocol. The cRNA was cleaned-up again using the GeneChip Sample Cleanup Module. Concentration and absorbance ratios of the cRNA was checked on the NanoDrop® ND-1000 Spectrophotometer and the quality evaluated on the 2100 Bioanalyzer for a smear of products ranging from 500–3000bps.

Labelled cRNA was fragmented by the MRC CSC Microarray Centre and each sample was hybridised to a GeneChip® Mouse Genome 430 2.0 Array (Affymetrix, USA) as specified by the manufacturer. Further details on the microarray hybridisation are available at the MRC/CSC/Imperial College Microarray Centre website (http://microarray.csc.mrc.ac.uk). Microarray data were analysed on the Rosetta Resolver® Gene Expression Analysis System (Rosetta Biosoftware, USA). Hybridisations or profiles for each sample in the TAG1 time course were grouped in an experimental definition and subjected to interchip normalization and nonlinear error correction to create ratio experiments. Log_10_ ratios between each sample in a time course were computed generating all possible pairwise comparisons of the time points. An error weighted average of the expression signal ratio and *P* value was calculated for each gene in each pairwise comparison. Changes in gene expression were considered as statistically significant if the calculated *P* value was equal to or below a threshold of 0.01.

The annotated information for each target genes is shown in Table S7. The information was compiled from the Mouse Genome Database http://www.informatics.jax.org/ [69], Entrez Gene http://www.ncbi.nlm.nih.gov/sites/entrez?db=gene [70] and Gene Ontology http://www.geneontology.org/ [71]. Gene expression in the different tissues and at different stages was curated from cDNA source data provided in the Mouse Genome Database.

### Embryo culture

For inhibition of Nodal/Smad2/3 signalling, embryos from CD1 inter-cross litters were dissected in ice cold PBS supplemented with 1% FCS on E5 or seventh E6 and cultured for 18 hours in 1∶1 DMEM: rat serum containing 20 µM of SB-431542 (Sigma) or 0.2% DMSO alone as a control in a 37°C, 5% CO_2_ incubator. Total RNA was extracted using Trizol® Reagent according to the manufacturer's protocol.

**Luciferase assays:** as described before [19]

**RT-PCR and Real-time qRT-PCR:** as described before [19]

Primer Sequences for housekeeping genes were described in [72]. Gene-specific primer sequences were obtained from PrimerBank (http://pga.mgh.harvard.edu/primerbank/index.html)

### Identification of ASE elements, CAGA boxes and multispecies sequence conservation

The sequences of interest were retrieved from NCBI or Ensembl. These included 10 kb upstream and downstream of the first and last exon. Potential ASE binding sites and CAGA boxes were identified using Fuzznuc, a program of the EMBOSS-MS software, which allows fuzzy searching of nucleic acid patterns using IUPAC codes and variable spacing between binding sites. Potential ASE binding sites were identified using the rule that two AATMMACA consensus sequences are separated by 30–200 bases, where M is C or A. In order to cover all possible patterns that correspond to an ASE element, the following rules were also tested: 1) TGTKKATT, 30–200 bp space followed by TGTKKATT (K representing T or G), 2) AATMMACA, 30–200 bp space followed by TGTKKATT or 3) TGTKKATT, 30–200 bp space followed by AATMMACA. Potential CAGA boxes were identified using the consensus AGMCAGACA or its reverse complement sequence TGTCTGKCT.

Multiple alignments of the genic sequences including 10 kb upstream of the first exon and 10 kb downstream of the last exon of genes that were predicted to contain ASE elements were generated and visualized using MULTIPIPMAKER (http://pipmaker.bx.psu.edu/pipmaker). In MULTIPIPMAKER, the reference mouse sequences were compared with the corresponding human, chimp, rat and dog genomic sequences in order to identify regions of high conservation across species. These regions were then manually inspected for the conservation of the predicted ASE elements.

## Supporting Information

Figure S1 Alk4* induction phosphorylates efficiently Smad2 and activates endogenous Nodal expression in TAG1 cells (A) Western blot analysis of total Smad2, P-Smad2, and PCNA (loading control) in TAG1 ES cells treated with: DMSO control medium, SB inhibitor (dissolved in DMSO), or Dox (dissolved in DMSO) for the time period indicated in hours. Bar chart represents densitometry analysis of the bands on the western bolt. P-Smad2 levels were normalised against total Smad2. All values are expressed relative to the DMSO control, which is represented as 100% on the chart. (B) Quantitative Real-Time PCR of Nodal transcripts in TAG1 cells at different time points (indicated in hours) are shown with blue line for DMSO treated cells, with red for SB, and with green for Dox. Relative transcript abundance is shown on the y-axis and time points on the x-axis, as indicated. All cells were pre-treated with SB for 6 hours (−6 +SB). Relative Nodal transcript abundance is expressed as the average of four PCR reactions (n = 4) normalised to the expression of the housekeeping genes: Gapdh, Ube, Ywhaz and B2m, with standard error the mean of the PCR reactions.
(3.74 MB TIF)

Figure S2 Validation of gene expression downstream Smad2/3 activation and inhibition in TAG1 ES cells. Semi-quantitative (A) and quantitative (B and C) RT-PCR for selected genes at different time points after Smad2/3 activation (+Dox) and inhibition (+SB), as indicated in hours. In (A) PCNA housekeeping gene expression was used as control gene; +, with reverse transcriptase; −, without. In (B) and (C) the relative transcript abundance was normalised to that of the housekeeping genes Gapdh, Ube, Ywhaz and B2m (y-axis) and shown at different time points (x-axis) during activation as indicated. The experiments were repeated three times with similar results (not shown).
(6.20 MB TIF)

Figure S3 Pipplots of the genomic sequence comparisons between mouse, human, chimp, rat and dog. For each gene, the reference sequence on top is the mouse sequence and the boxes underneath represent the corresponding sequences in the other species. Short black lines in the rectangles represent sequence similarities greater than 50% between the reference and the other species. The presence and position of the ASE elements in the sequence comparisons is illustrated by the red rectangles.
(3.43 MB TIF)

Figure S4 Sequence conservation of the ASE enhancer elements in known and novel Smad2/3 target genes. A black dot indicates conservation of a base between the reference (mouse) and the other species, while an alternative base indicates the difference. The ASE elements are enclosed in red rectangles. In (A), the predicted ASE element in the genic sequence of Nodal, a known Smad2/3 target, is conserved in all species tested; the ASE element in Ubr7 gene shows medium conservation only in rodents; for the CD97 gene, one ASE is not conserved and the other conserved only in rodents. The predicted ASE elements in the genic sequence of the known Smad2/3 target genes Pitx2, Lefty1 and Lefty2 is conserved in all species tested (B). The first predicted ASE elements in the genic sequence of Zfp423 gene is conserved in all species tested, while the remaining ASE elements have been modified by insertions (second ASE), point mutations of important nucleotides (third and fourth ASE) or deletions (fifth ASE) in the human and chimp (C).
(10.16 MB EPS)

Table S1 Behaviour and classification of gene expression in the Dox/SB15 experiment.
(0.09 MB PDF)

Table S2 Behaviour and classification of gene expression in the Dox/SB12 experiment
(0.07 MB PDF)

Table S3 Upregulated genes downstream Smad2/3 activation in the absence of protein synthesis
(0.06 MB PDF)

Table S4 Analysis by Fuzznuc for the existence of ASE elements and CAGA boxes Summary of the genomic coordinates, exact binding site and position of ASE elements with regard to the gene start. Genes that did not show significant fold-change in the absence of protein synthesis (§) and are used as controls. bp (base pairs); Ch (chromosome)
(0.02 MB XLS)

Table S5 Behaviour of selected known Nodal-regulated genes in TAG1 ES cells during Smad2/3 activation in the absence of protein synthesis
(0.06 MB PDF)

Table S6 Behaviour of selected known Nodal-regulated genes in TAG1 ES cells during Smad2/3 activation and repression in the presence of protein synthesis
(0.07 MB PDF)

Table S7 Functional annotation of Smad2/3 target genes
(0.11 MB PDF)
