# Correction: Graded Smad2/3 Activation Is Converted Directly into Levels of Target Gene Expression in Embryonic Stem Cells

**DOI:** 10.1371/annotation/8f64f0c8-6767-4701-8ded-7f937d953cb6

**Published:** 2009-02-09

**Authors:** Marcela Guzman-Ayala, Kian Leong Lee, Konstantinos J. Mavrakis, Paraskevi Goggolidou, Dominic P. Norris, Vasso Episkopou

The link to the microarray data was mistakenly omitted. The data can be found at: http://www.ebi.ac.uk/microarray-as/ae/browse.html?keywords=E-TABM-574

