# Supplementary material for: Graded Smad2/3 Activation Is Converted Directly into Levels of Target Gene Expression in Embryonic Stem Cells
Source: PLoS One. 2009 Jan 27;4(1):e4268. doi: 10.1371/journal.pone.0004268 (PMC2627943; doi:10.1371/journal.pone.0004268)
Supplement: Table S1 — Behaviour and classification of gene expression in the Dox/SB15 experiment. (0.09 MB PDF) [file pone.0004268.s005.pdf]

Table S1. Behaviour and classification of gene expression in the Dox/SB15 experiment.

High Response Targets (10- to 100-fold)

			Induction and inhibition* of TAG1 Cells					Dox treatment of J1 parental cells	
**Gene**	**Probe Sets ID**	**Accession**	**0h-6h**	**0h-12h**	**0h-15h***	**0h-18h**	**0h-21h**	**0h-6h**	**0h12h**
Pitx2	1424797_a_at	U80011	64.469	100	100	59.390	29.398	1.112	1.572
Lefty1	1417638_at	NM_010094	33.096	39.434	51.985	9.492	6.470	1.351	2.215
GalNAcS-6ST	1452092_at	AK019474	11.740	21.509	36.318	4.613	5.456	4.162	7.34
Lefty2	1436227_at	AV214969	19.378	19.628	22.692	11.832	5.450	-1.001	-1.008
Pitx2	1450482_a_at	AB006320	9.427	14.620	20.173	7.640	4.568	1.29	1.519
Fgf15	1418376_at	NM_008003	8.118	14.712	15.146	4.657	7.666	2.226	3.16
Acvr1b	1422098_at	NM_007395	13.055	10.172	13.759	6.473	1.368	-1.319	-1.266

Medium Response Targets (2.5- to 10-fold)

			Induction and inhibition* of TAG1 Cells					Dox treatment of J1 parental cells	
**Gene**	**Probe Sets ID**	**Accession**	**0h-6h**	**0h-12h**	**0h-15h***	**0h-18h**	**0h-21h**	**0h-6h**	**0h-12h**
1443256_at	1443256_at	BB548833	3.537	4.699	8.196	5.607	5.175	1.651	2.237
Pcdh8	1447825_x_at	BB076893	2.208	5.998	7.615	3.257	4.729	1.394	1.762
AW548124	1454838_s_at	BB323985	3.886	7.797	7.440	2.988	2.136	-1.559	-1.139
Duxbl	1445710_x_at	AV321065	1.995	5.154	5.521	3.627	4.768	1.858	3.497
Smad7	1423389_at	BF226166	6.848	6.332	4.977	1.625	1.848	-1.087	-1.277
Tmem63a	1423871_at	BC019442	3.864	3.120	4.956	3.099	1.866	-1.204	1.112
Smad7	1443771_x_at	BB241324	5.239	5.791	4.792	1.982	1.771	-1.015	-1.133
AW548124	1460411_s_at	BC022157	2.656	4.592	4.593	2.286	1.696	-1.305	-1.078
Tmepai	1422706_at	AV370981	3.896	3.256	4.066	1.750	-1.040	1.129	-1.058
Cnpy1	1437996_s_at	BB131676	2.029	2.489	3.944	2.544	2.118	1.498	1.74
Nxn	1422465_a_at	BB366804	2.019	4.341	3.674	2.886	3.094	1.1	1.689
Bcar3	1415936_at	NM_013867	2.703	3.671	3.315	2.295	2.352	1.282	1.454
Pycr2	1448315_a_at	NM_133705	2.633	2.897	3.314	2.844	1.782	1.016	-1.034
Cd97	1418394_a_at	NM_011925	2.131	2.958	3.044	2.639	1.714	1.22	1.48
Plekha2	1417288_at	NM_031257	1.939	3.016	2.883	2.297	1.695	1.006	1.035
Slc7a7	1447181_s_at	AI790233	1.854	2.821	2.727	1.905	2.362	1.239	1.552
Lgr4	1433891_at	BI107632	1.964	2.274	2.625	2.266	1.637	1.072	1.171
Camk2n1	1456609_at	BE994488	2.324	1.597	2.583	1.105	1.601	1.054	1.356
Slc7a7	1417392_a_at	NM_011405	1.914	2.720	2.578	1.821	2.243	1.215	1.565
**Eif2c1**	**1434331_at**	**BG072783**	**2.678**	**2.493**	**2.573**	**1.752**	**2.474**	**1.887**	**2.313**

Low Response Targets (1.2- to 2.5-fold)

			Induction and inhibition* of TAG1 Cells					Dox treatment of J1 parental cells	
**Gene**	**Probe Sets ID**	**Accession**	**0h-6h**	**0h-12h**	**0h-15h***	**0h-18h**	**0h-21h**	**0h-6h**	**0h-12h**
Abcg2	1422906_at	NM_011920	1.882	2.608	2.386	1.912	1.597	1.152	1.223
SnoN	1422054_a_at	U36203	2.108	2.579	2.355	1.423	2.227	1.507	1.855
Rasd2	1427344_s_at	BC026377	1.444	1.463	2.331	1.694	1.152	-1.286	-1.091
Dusp9	1433845_x_at	AV295798	1.618	2.190	2.316	2.270	2.058	1.053	1.306
Bhlhb8	1449233_at	BC011486	1.160	2.393	2.292	1.810	1.897	1.548	1.966
Sntb2	1449840_at	BI646094	2.237	1.920	2.260	1.842	1.285	-1.04	-1.063
Dusp9	1454737_at	AV295798	1.642	2.157	2.236	2.119	1.770	-1.021	1.054
Nphs1	1422142_at	AF172256	1.361	2.306	2.225	1.803	1.744	1.267	1.634
Nodal	1422057_at	X70514	1.275	1.415	2.138	-1.037	-1.243	-1.029	1.088
SnoN	1452214_at	AK018608	1.821	2.051	2.041	1.678	1.927	-1.011	1.532
Notch3	1421965_s_at	NM_008716	1.413	1.990	2.027	1.925	1.675	-1.091	-1.043

Tmepai	1422705_at	AV370981	2.438	1.698	1.995	-1.287	-1.150	1.071	1.235
D6Wsu176e	1417953_at	AK016470	1.557	1.928	1.994	1.760	1.551	1.319	1.28
Ubr7	1433479_at	AV030071	1.442	1.830	1.923	1.713	1.472	1.015	1.188
Pea15	1416407_at	AI323543	1.370	1.617	1.908	1.683	1.449	1.023	1.227
Gpr107	1454616_at	AV030071	1.527	1.905	1.863	1.600	1.375	1.215	1.436
Cripto	1450989_at	AV294613	1.607	2.026	1.841	1.532	1.706	-1.303	-1.372
Bbc3	1423315_at	AW489168	1.120	1.337	1.826	1.834	1.073	-1.063	-1.043
Ubr7	1459788_at	BB115649	1.479	1.503	1.761	1.601	-1.010	-1.84	-1.874
Schip1	1423025_a_at	NM_013928	1.342	1.499	1.761	1.712	1.349	-1.077	-1.011
Epha2	1421151_a_at	NM_010139	1.783	1.747	1.725	1.157	1.405	-1.172	-1.405
Ppp1r2	1417341_a_at	NM_025800	1.301	1.467	1.651	1.609	1.382	1.148	1.185
Rhob	1449110_at	BC018275	1.365	1.281	1.636	1.146	1.224	-1.259	-1.295
Ski	1429192_at	AV381512	1.509	1.280	1.625	1.525	1.214	1.086	-1.047
Zcchc11	1437395_at	BE370775	1.240	1.474	1.596	1.564	1.355	1.018	1.137
Atrx	1420948_s_at	BB825830	1.215	1.458	1.562	1.423	1.206	1.043	1.087
**Tmepai**	**1438783_at**	**BB325257**	**1.692**	**1.317**	**1.557**	**-1.945**	**-1.849**	**4.361**	**2.522**
Mcl1	1416881_at	BC003839	1.219	1.298	1.549	1.097	1.308	1.082	1.046
Zfp423	1419380_at	NM_033327	1.075	1.441	1.535	-1.052	1.032	-1.041	1.135
Ccnd2	1416122_at	NM_009829	1.607	1.899	1.501	-1.120	-1.582	-1.117	1.064
Eif3s6ip	1437948_x_at	BB443362	1.161	1.357	1.499	1.400	-1.067	-1.481	-1.386
**Tex19**	**1417482_at**	**NM_028602**	**1.258**	**1.364**	**1.486**	**1.375**	**1.095**	**1.249**	**1.386**
**Dppa2**	**1429654_at**	**AK010743**	**1.339**	**1.481**	**1.462**	**1.263**	**1.371**	**1.232**	**1.622**
Ccnd2	1434745_at	BQ175880	1.541	1.841	1.438	-1.241	-1.524	-1.031	1.141
5730419I09 Rik	1437003_at	BB323930	1.982	1.391	1.424	1.430	-1.018	1.112	1.08
Aasdhppt	1428757_at	AK013111	1.204	1.347	1.416	1.377	1.127	1.006	-1.043
Dppa2	1453223_s_at	AK010743	1.182	1.431	1.415	1.270	1.327	1.068	1.112
**Llglh2**	**1423938_at**	**AY033650**	**1.165**	**1.429**	**1.359**	**1.307**	**1.041**	**1.203**	**1.404**
Fbxl20	1456378_s_at	AV120094	1.343	1.326	1.356	1.524	1.110	-1.076	-1.102
Moap1	1448787_at	BC014715	1.099	1.332	1.346	-1.023	1.132	1.02	1.076
B3galt3	1418736_at	BC003835	1.957	1.441	1.343	-1.186	-1.839	-1.333	-1.844
Khsrp	1436813_x_at	BB332580	1.311	1.240	1.336	1.401	-1.043	-1.338	-1.26
BC037674	1434835_at	BM230523	1.219	1.207	1.309	1.115	1.007	1.169	1.134
Mrpl15	1430798_x_at	AV306676	1.037	1.095	1.307	1.042	-1.338	-1.311	-1.473
D030056L22 Rik	1423879_at	BC020125	1.388	1.339	1.304	1.236	1.040	1.013	-1.018
Ttc13	1437709_x_at	BB492914	1.202	1.406	1.270	1.227	1.017	1.052	1.106
Nfkbia	1420088_at	AI462015	1.209	1.144	1.225	1.053	-1.081	-1.097	-1.229
Hrb	1426923_at	BB130716	1.420	1.164	1.215	1.106	-1.020	1.092	1.034

Values represent the fold change in gene expression at each time point compared to the 0 hours (0h) uninduced control. Upregulation of gene expression occurs at the time points corresponding to induction (0h-6h, 0h-12h and 0h-15h) whereas subsequent SB treatment at 15h results in the downregulation of the same genes (0h-18h and 0h-21h). Genes significantly upregulated in J1 parental cell line are highlighted in bold. Classification is done based on the fold induction at 15 hours.
