# Supplementary material for: Graded Smad2/3 Activation Is Converted Directly into Levels of Target Gene Expression in Embryonic Stem Cells
Source: PLoS One. 2009 Jan 27;4(1):e4268. doi: 10.1371/journal.pone.0004268 (PMC2627943; doi:10.1371/journal.pone.0004268)
Supplement: Table S2 — Behaviour and classification of gene expression in the Dox/SB12 experiment (0.07 MB PDF) [file pone.0004268.s006.pdf]

Table S2. Behaviour and classification of gene expression in the Dox/SB12 experiment

High Response Targets (Replicates, 10.0- to 100.0-fold)

Gene	Probe Sets ID	Accession	0h-6h	0h-12h	0h-15h	0h-21h
Lefty1	1417638_at	NM_010094	100.000	100.000	100.000	38.219
Lefty2	1436227_at	AV214969	34.106	39.454	33.128	12.572
Pitx2	1424797_a_at	U80011	13.330	27.074	23.163	5.868
Fgf15	1418376_at	NM_008003	21.403	25.922	10.576	8.451
**Bhlhb8**	**1449233_at**	**BC011486**	**4.992**	**14.738**	**10.817**	**9.863**

Medium Response Targets (Replicates, 2.5- to 10.0-fold)

Gene	Probe Sets ID	Accession	0h-6h	0h-12h	0h-15h	0h-21h
Pcdh8	1447825_x_at	BB076893	3.021	9.422	7.919	8.178
**Pitx2**	**1450482_a_at**	**AB006320**	**7.253**	**8.741**	**7.424**	**2.295**
**GalNAcS-6ST**	**1452092_at**	**AK019474**	**2.556**	**7.839**	**7.327**	**-1.056**
AW548124	1454838_s_at	BB323985	2.085	7.404	5.197	3.860
Duxbl	1445710_x_at	AV321065	3.116	6.338	7.224	4.140
Smad7	1423389_at	BF226166	6.029	5.680	1.777	1.915
AW548124	1460411_s_at	BC022157	4.078	4.685	3.703	2.016
Smad7	1443771_x_at	BB241324	2.466	4.492	1.901	2.089
**Gpr107**	**1459788_at**	**BB115649**	**1.747**	**4.354**	**3.512**	**4.980**
Slc7a7	1447181_s_at	AI790233	2.318	4.266	3.343	2.895
Plekha2	1417288_at	NM_031257	2.367	3.876	3.172	1.749
Bcar3	1415936_at	NM_013867	3.151	3.672	3.173	2.506
**Notch3**	**1421965_s_at**	**NM_008716**	**1.415**	**3.509**	**2.919**	**2.708**
SnoN	1422054_a_at	U36203	2.393	3.459	2.391	2.629
**Abcg2**	**1422906_at**	**NM_011920**	**2.386**	**3.419**	**2.932**	**2.352**
1443256_at	1443256_at	BB548833	2.227	3.309	3.556	1.994
Slc7a7	1417392_a_at	NM_011405	2.088	3.251	2.718	2.346
Tmem63a	1423871_at	BC019442	2.388	3.176	3.259	1.474
**Fbxl20**	**1456378_s_at**	**AV120094**	**1.002**	**2.883**	**2.500**	**3.244**
**Epha2**	**1421151_a_at**	**NM_010139**	**2.025**	**2.843**	**1.588**	**1.554**
Nxn	1422465_a_at	BB366804	2.038	2.722	2.399	1.411
Pycr2	1448315_a_at	NM_133705	2.797	2.692	2.676	1.480
**Cripto**	**1450989_at**	**AV294613**	**1.444**	**2.572**	**1.996**	**2.968**

Low Response Targets (Replicates, 1.0- to 2.5-fold)

Gene	Probe Sets ID	Accession	0h-6h	0h-12h	0h-15h	0h-21h
Sntb2	1449840_at	BI646094	1.768	2.349	2.083	2.233
Nphs1	1422142_at	AF172256	1.605	2.293	2.114	1.403
D6Wsu176e	1417953_at	AK016470	1.681	2.195	2.054	1.623
D030056L22Rik	1423879_at	BC020125	1.235	2.194	1.842	2.475
**Tmepai**	**1422706_at**	**AV370981**	**3.704**	**2.178**	**1.673**	**1.070**
Atrx	1420948_s_at	BB825830	1.141	2.172	1.946	1.971
**Cnpy1**	**1437996_s_at**	**BB131676**	**1.053**	**2.091**	**1.839**	**1.376**
Zcchc11	1437395_at	BE370775	1.245	2.050	1.810	2.374
Ccnd2	1434745_at	BQ175880	1.559	2.018	1.763	-1.258
Rhob	1449110_at	BC018275	1.445	2.018	2.050	1.715
Zfp423	1419380_at	NM_033327	1.024	2.015	1.727	1.142
Ubr7	1433479_at	AV030071	1.647	2.015	1.977	1.344
Ubr7	1454616_at	AV030071	1.647	2.015	1.977	1.344
Bbc3	1423315_at	AW489168	1.209	1.999	2.270	1.558

**Camk2n1**	**1456609_at**	**BE994488**	**1.719**	**1.908**	**1.742**	**1.167**
Mcl1	1416881_at	BC003839	1.313	1.904	1.984	1.515
Pea15	1416407_at	AI323543	1.128	1.850	1.744	1.530
SnoN	1452214_at	AK018608	2.680	1.827	1.457	2.094
Dusp9	1433845_x_at	AV295798	1.486	1.770	1.641	1.475
Nfkbia	1420088_at	AI462015	1.330	1.769	1.724	1.446
Dusp9	1454737_at	AV295798	1.807	1.758	1.710	1.448
**Cd97**	**1418394_a_at**	**NM_011925**	**2.960**	**1.731**	**1.562**	**-1.178**
Rasd2	1427344_s_at	BC026377	2.033	1.714	1.826	1.616
Dppa2	1453223_s_at	AK010743	1.250	1.688	1.492	1.662
Ski	1429192_at	AV381512	1.096	1.686	1.637	1.254
Hrb	1426923_at	BB130716	1.505	1.671	1.652	1.515
Schip1	1423025_a_at	NM_013928	1.366	1.655	1.776	1.522
B3galt3	1418736_at	BC003835	1.644	1.643	1.615	1.056
**Lgr4**	**1433891_at**	**BI107632**	**2.530**	**1.625**	**1.481**	**1.667**
Eif3s6ip	1437948_x_at	BB443362	1.532	1.613	1.512	1.872
BC037674	1434835_at	BM230523	1.363	1.606	1.624	1.343
Moap1	1448787_at	BC014715	1.365	1.574	1.369	1.009
Ccnd2	1416122_at	NM_009829	1.936	1.549	1.466	-1.541
Mrpl15	1430798_x_at	AV306676	1.443	1.438	1.548	1.284
Ppp1r2	1417341_a_at	NM_025800	1.079	1.421	1.533	1.400
Aasdhppt	1428757_at	AK013111	1.283	1.418	1.575	1.453
5730419I09Rik	1437003_at	BB323930	1.470	1.356	1.158	1.211
Ttc13	1437709_x_at	BB492914	1.119	1.351	1.210	1.367
Nodal	1422057_at	X70514	1.268	1.182	-1.164	-2.980
Khsrp	1436813_x_at	BB332580	1.383	1.061	1.001	1.246

Values represent the fold change in gene expression at each time point compared to the 0 hours (0h; uninduced control). Changes in gene expression are shown for all intermediate time points. Upregulation of gene expression occurs at time points corresponding to induction (0h-6h and 0h-12h), whereas subsequent inhibition at 12h results in the downregulation of the same genes (0h-15h and 0h-21h). Genes in bold exhibit different sensitivity in the Dox/SB15 experiment (Table S1).
