# Supplementary material for: Graded Smad2/3 Activation Is Converted Directly into Levels of Target Gene Expression in Embryonic Stem Cells
Source: PLoS One. 2009 Jan 27;4(1):e4268. doi: 10.1371/journal.pone.0004268 (PMC2627943; doi:10.1371/journal.pone.0004268)
Supplement: Table S3 — Upregulated genes downstream Smad2/3 activation in the absence of protein synthesis (0.06 MB PDF) [file pone.0004268.s007.pdf]

Table S3. Upregulated genes downstream Smad2/3 activation in the absence of protein synthesis

Gene	Fold-change of expression				
	0h-2h	0h-4h	0h-6h	0h-8h	0h-12h
*Fgf15	1.651	1.546	1.390	-1.366	-1.544
*Acvr1b	1.575	1.331	1.624	1.233	-1.639
Slc7a7	1.147	1.197	1.285	1.209	-1.018
Hrb	1.075	1.181	1.352	1.14	1.278
Bbc3	1.302	1.13	-1.25	-1.247	1.193
Ski	1.186	1.107	-1.558	-2.228	-3.408
Nphs1	-1.12	1.1	-1.822	-1.917	-1.708
Abcg2	1.057	1.089	1.201	1.189	-1.166
B3galt3	-1.071	1.083	-1.732	-2.715	-1.455
Dppa2	1.086	1.059	-1.036	-1.058	-1.167
Cripto	-1.104	1.026	-2.213	-2.605	-7.238
Ttc13	-1.124	1.018	-1.153	-1.215	-1.206
Sntb2	1.065	1.018	-1.196	-1.678	-1.849
Ppp1r2	1.016	1.009	-1.192	1.026	-1.022
Aasdhppt	-1.084	1	-1.028	1.016	-1.129
5730419I09Rik	1.061	-1.002	-1.244	-1.414	-1.857
Notch3	-1.142	-1.006	-1.064	1.086	-1.076
Bhlhb8	-1.175	-1.006	1.208	-1.142	-1.743
D6Wsu176e	1.107	-1.015	1.13	1.058	-1.428
Lgr4	1.13	-1.017	-1.616	-1.695	-1.649
Pea15	-1.027	-1.041	1.123	1.028	1.126
Dusp9	1.006	-1.048	1.023	-1.016	-1.077
Mrpl15	1.131	-1.053	-1.621	-2.087	-3.101
Ccnd2	-1.034	-1.059	-2.189	-2.807	-4.274
BC037674	-1.016	-1.071	1.033	1.01	1.018
Khsrp	-1.101	-1.074	-1.53	-1.356	-1.43
Eif3s6ip	-1.284	-1.089	-1.426	-1.887	-1.241
Dusp9	1.05	-1.105	1.19	1.062	-1.074
Ccnd2	-1.004	-1.203	-1.473	-1.915	-2.728
Atrx	-1.334	-1.252	-1.768	-1.529	-1.588
Grp107	-1.194	-1.286	-1.31	-1.521	-1.56

Fold-change of expression for specified target genes at indicated time points after removal of SB and Smad2/3 activation in the absence of protein synthesis compared to the time point 0 hours. At time point 0 the TAG1 cells have been pre-treated with SB for 6 hours and with SB + Dox for an additional 6 hours. Genes are classified in descending order with the genes showing upregulation >1.2-fold 4 hours after activation of Smad2/3. Genes with * are also upregulated by rtTA/Dox (Table S1)
