# Supplementary material for: Graded Smad2/3 Activation Is Converted Directly into Levels of Target Gene Expression in Embryonic Stem Cells
Source: PLoS One. 2009 Jan 27;4(1):e4268. doi: 10.1371/journal.pone.0004268 (PMC2627943; doi:10.1371/journal.pone.0004268)
Supplement: Table S5 — Behaviour of selected known Nodal-regulated genes in TAG1 ES cells during Smad2/3 activation in the absence of protein synthesis (0.06 MB PDF) [file pone.0004268.s009.pdf]

Table S5. Behaviour of selected known Nodal-regulated genes in TAG1 ES cells during Smad2/3 activation in the absence of protein synthesis

Upregulated genes in the absence of protein synthesis

Sequence ID	Gene	0h-2h	0h-4h	0h-6h	0h-8h	0h-12h
730473	Gadd45g	10.837	18.769	23.901	27.894	24.330
728665	Omd	4.967	2.436	2.865	2.601	4.046
722614	Gadd45b	4.925	8.338	11.788	12.835	10.478
719641	Arl4a	3.991	3.231	2.727	2.715	4.791
731906	Id3	2.737	5.266	5.049	4.753	2.630
746082	E130016E03Rik	2.590	1.273	2.892	3.745	2.241
727593	Sell	2.454	1.735	1.834	1.009	2.847
745899	Dkk1	2.223	2.520	1.891	2.278	1.707
752900	Otx1	2.171	3.865	2.737	2.621	1.952
722639	Fzd8	1.934	4.530	2.779	2.239	-1.783
729715	Hist1h2bc	1.794	6.311	8.318	9.164	12.337
722051	Bambi	1.778	2.908	3.097	1.573	2.160
724753	Lhx1	1.743	3.370	2.194	3.315	2.490
729638	Myh11	1.674	1.379	1.567	2.004	1.637
723437	Foxa2	1.664	2.727	3.203	2.086	1.580
730508	Snai1	1.469	2.150	1.855	1.855	1.563
719620	Gata6	1.439	1.206	1.261	-1.079	-1.246
731300	Cdc6	1.438	1.964	1.823	1.679	1.521
721276	Arhgef3	1.428	1.990	1.398	1.207	-1.104
725125	Sox17	1.405	1.445	1.413	1.308	-1.006
730886	Chrd	1.386	1.204	1.770	1.387	1.481
761537	Dhrs13	1.370	1.446	1.349	1.403	-1.046
760469	Zcchc4	1.369	1.830	1.521	1.861	2.362
747976	Syt7	1.362	2.417	2.210	1.963	1.159
727317	Efnb2	1.345	1.993	1.939	1.185	1.489
725985	Gata5	1.335	1.443	1.440	1.241	1.399
730477	Sart3	1.320	1.527	1.737	1.641	1.693
732720	Zfand5	1.319	1.234	1.066	1.043	1.151
727719	Serpinb11	1.308	1.478	1.557	1.428	1.790
727401	Efnb2	1.291	2.717	2.102	1.561	1.411
722270	Rasd1	1.266	1.486	-1.081	1.212	-1.477
731120	Arl4a	1.259	1.443	1.395	1.097	-1.089
720521	Slc25a28	1.220	1.315	1.066	-1.227	-2.042
722702	Hhex	1.207	1.230	1.285	1.077	-1.188

Fold-change of gene expression in TAG1 ES cells at different time points compared to the uninduced time point 0h. Genes are listed from top to bottom according to their fold-change at time point 2h.
