# Supplementary material for: Graded Smad2/3 Activation Is Converted Directly into Levels of Target Gene Expression in Embryonic Stem Cells
Source: PLoS One. 2009 Jan 27;4(1):e4268. doi: 10.1371/journal.pone.0004268 (PMC2627943; doi:10.1371/journal.pone.0004268)
Supplement: Table S6 — Behaviour of selected known Nodal-regulated genes in TAG1 ES cells during Smad2/3 activation and repression in the presence of protein synthesis (0.07 MB PDF) [file pone.0004268.s010.pdf]

Table S6. Behaviour of selected known Nodal-regulated genes in TAG1 ES cells during Smad2/3 activation and repression in the presence of protein synthesis

Genes with significant fold-change in Dox/SB15 experiment at time point 6 hours

Sequence ID	Gene	0h-6h	0h-12h	0h-15h	15h-18h	15h-21h
730473	Gadd45g	2.686	4.704	3.775	-1.501	1.742
734743	Sox17	2.673	2.680	3.376	-1.670	-1.063
723437	Foxa2	2.184	-1.069	3.990	-1.529	-1.191
725985	Gata5	2.009	2.429	1.841	1.281	-1.731
728665	Omd	1.942	1.358	-1.085	-1.770	-1.243
741542	Syt7	1.868	1.789	1.962	-1.209	-2.227
750492	Efnb2	1.638	2.009	1.984	-1.391	-1.175
746082	E130016E03Rik	1.463	1.790	1.830	1.016	1.076
745899	Dkk1	1.455	1.662	1.293	1.368	-1.599
722051	Bambi	1.438	1.230	1.519	-1.454	-1.581
724753	Lhx1	1.425	1.498	2.475	-1.314	-1.931
755379	E130016E03Rik	1.378	1.202	1.140	-1.221	1.090
749153	Zfand5	1.224	1.270	-1.057	1.246	1.089
727316	Efnb2	1.207	1.273	1.831	-1.182	-1.153

Genes with significant fold-change in Dox/SB12 experiment at time point 6 hours

Sequence ID	Gene	0h-6h	0h-12h	12h-15h	15h-21h
741542	Syt7	3.268	2.797	-1.186	-1.152
723437	Foxa2	2.750	3.182	-1.230	1.053
727401	Efnb2	2.282	2.309	1.042	1.036
729638	Myh11	2.036	2.035	1.723	1.462
728665	Omd	1.965	1.073	1.271	-2.316
730886	Chrd	1.853	2.821	1.483	1.196
727593	Sell	1.794	2.087	-6.657	-5.157
749153	Zfand5	1.775	-1.380	-1.001	-1.133
734743	Sox17	1.655	-2.004	1.469	1.307
746482	Gata6	1.638	-1.285	-1.528	-1.317
723163	Syt7	1.550	1.525	1.420	1.519
736989	Gadd45g	1.517	2.381	2.492	1.154
747690	Syt7	1.513	-1.012	1.242	-1.024
724753	Lhx1	1.496	1.683	-2.408	-1.853
729637	Myh11	1.464	2.245	1.966	-1.065
760116	Zfand5	1.365	2.097	1.174	-1.328
752900	Otx1	1.346	-1.242	2.693	3.443
745899	Dkk1	1.299	-1.408	-3.734	-1.105
722051	Bambi	1.294	1.187	1.430	1.060
719641	Arl4a	1.292	1.034	-1.954	1.244
725985	Gata5	1.269	-1.398	-1.403	-3.483
730473	Gadd45g	1.243	4.021	-2.376	-1.262

Fold-change of gene expression in TAG1 ES cells at different time points after Alk4* induction compared to the uninduced time point 0h, and under repression compared to the maximum time of induction in the two different experiments, Dox12 and Dox15 (time point 12h or 15 h respectively). Genes are listed from top to bottom according to their fold-change at the first time point upon Smad2/3 phosphorylation (6 hours).
