# Supplementary material for: Graded Smad2/3 Activation Is Converted Directly into Levels of Target Gene Expression in Embryonic Stem Cells
Source: PLoS One. 2009 Jan 27;4(1):e4268. doi: 10.1371/journal.pone.0004268 (PMC2627943; doi:10.1371/journal.pone.0004268)
Supplement: Table S7 — Functional annotation of Smad2/3 target genes (0.11 MB PDF) [file pone.0004268.s011.pdf]

Table S7. Functional annotation of Smad2/3 target genes

Genes upregulated in the absence of protein synthesis are marked with *

Transcription

symbol	ontology	component	expression
Atrx (XH2, Xnp, ATR2, MRXS3, Rad54, Hp1bp2, ZNF-HX, Hp1bp38, DXHXS6677E)	ATP binding, ATP-dependent helicase activity, chromatin binding, hydrolase activity, metal ion binding, nucleic acid binding, protein binding, zinc ion binding/ DNA repair, forebrain development, response to DNA damage stimulus	heterochromatin, nuclear chromosome, nucleus	widely expressed
Khsrp (6330409F21Rik, KSRP)	ATP binding, DNA binding, RNA binding, aminoacyl-tRNA ligase activity, protein binding/ mRNA export from nucleus, mRNA processing, nuclear mRNA splicing, via spliceosome, tRNA aminoacylation for protein translation	nucleus	widely expressed
*Pitx2 (Brx1, Ptx2, Rieg, Brx1a, Brx1b, Otlx2, Munc30, Pitx2a, Pitx2b, Pitx2c, solurshin, 9430085M16Rik)	DNA binding, protein binding, sequence-specific DNA binding, transcription factor activity, transcription factor binding/ Wnt receptor signalling pathway, determination of left/right symmetry, extraocular skeletal muscle development (sensu Mammalia), heart development, pattern specification, patterning of blood vessels, regulation of cell migration, regulation of cell proliferation	nucleus, transcription factor complex	embryonic (lateral mesoderm, brain, eye, mandible, maxilla); postnatal (molar, pituitary gland, testis)
Ski (MGC8300, AA062172, AA589460, BC004088, 2310012I02Rik, 2610001A11Rik)	TGFb signalling, transcriptional repressor complex, cell differentiation, neural tube and skeletal muscle development, proto-oncogene,	cytoplasm, nucleus	widely expressed
*SnoN (Skil, sno, Skir, SnoN2, sno-dE3, 9130011J04Rik)	TGFb signalling, transcriptional repressor complex, cell differentiation, lymphocytes development, proto-oncogene	cytoplasm, nucleus,	widely expressed
*Zcchc11 (PPAPD3, KIAA0191, mKIAA0191, 6030404K05Rik)	nucleotidyltransferase activity, nucleic acid binding, nucleotidyltransferase activity, zinc ion binding/ cytokine production, inhibition of NF-kappaB transcription factor, regulation of lipopolysaccharide-mediated signalling pathway	nucleus, cytoplasm	embryonic (testis, brain); postnatal (lymph node, thymus); mammary gland tumour
*Zfp423 (Ebfaz, mKIAA0760, Roaz, Zfp104)	DNA binding, zinc ion binding, cell differentiation, multicellular organismal development, nervous system development	nucleus	embryonic (limb bud, mandible, ovary, uterus)

Signalling

symbol	ontology	component	expression
*Camk2n1 (1810006K23Rik)	protein kinase inhibitor activity, postynaptic density	synaptosome	postnatal (corpus striatum, pancreas)
Gpr107 (AI790205, mKIAA1624, C530034M11)	G-protein, receptor activity	integral to membrane, membrane	embryonic (eye, spinal cord, stomach); postnatal (brain, thymus, kidney)
*AW548124 (ESTM17, X83346, AI115348, MGC99930)	ATP binding, nucleotide binding, protein-tyrosine kinase activity		widely expressed
*Bcar3 (AND-34, AI131758)	GTPase mediated signal transduction, guanyl-nucleotide exchange factor activity/ intracellular signalling cascade	intracellular	widely expressed
Bhlhb8 (Mist1, 1810009C13Rik)	G-protein coupled receptor protein signalling pathway, DNA binding, protein binding, protein homodimerization activity, Golgi organization and biogenesis, calcium-mediated signalling, cell maturation, cell-cell signalling, glucose homeostasis, mitochondrial calcium ion transport, positive regulation of transcription from RNA polymerase II promoter	nucleus	widely expressed
Ccnd2 (Vin1, Vin-1, C86853, AI256817, BF642806, 2600016F06Rik)	cyclin-dependent protein kinase regulator activity, protein binding, cell division, regulation of progression through cell cycle	nucleus	widely expressed

*Cd97 (EGF-TM7 receptor, TM7LN1, AA409984)	G-protein coupled receptor activity, calcium ion binding, protein binding, signal transducer activity, transmembrane, cell adhesion, neuropeptide signalling pathway, signal transduction	extracellular space, integral to membrane, membrane	widely expressed
Dusp9 (Mpk4, Dusp4, Pyst3)	Map kinase tyrosine/serine/threonine phosphatase activity/ inactivation of MAP activity	cytoplasm	widely expressed
D6Wsu176e (Fam3c)	cytokine activity	extracellular region	embryonic (16-cell stage, ectoplacental cone, head)
*Epha2 (Eck, Myk2, Sek2, Sek-2, AW545284)	ephrin receptor signalling pathway, ATP binding, nucleotide binding, protein serine/threonine kinase activity, protein-tyrosine kinase activity, transferase activity, neuron differentiation, protein amino acid phosphorylation	extracellular space, integral to membrane, membrane	widely expressed
Fgf15	fibroblast growth factor receptor binding, growth factor activity/ heart development, neural crest cell migration, signal transduction	extracellular space	embryonic (brain, eye, retina, otocyst); lung and mammary gland tumour
*Lefty1 (Leftb, Stra3, Tgfb4, lefty, lefty-1, AI450052)	TGFb signalling, cytokine activity, growth factor activity, anterior-posterior axis specification, cell growth, cell migration during gastrulation, development, negative regulation of cell proliferation	extracellular space	embryonic (primitive endoderm, visceral endoderm, primitive streak, lateral plate mesenchyme, floor plate, neural tube, pancreas); postnatal (B-lymphocyte, lymph node, colon, mammary gland) mammary gland and lung tumour
*Lefty2 (Ebaf, Lefta, AV214969, MGC98569, 6030463A22Rik, left-right determination, factor A)	TGFb signalling, growth factor activity, cell growth, growth, axial patterning		embryonic (primitive streak, mesoderm, ectoderm, lateral plate mesenchyme, floor plate, germ cells, neural tube)
Lgr4 (Gpr48, 9130225G07, A930009A08Rik)	G-protein coupled receptor activity, signal transducer activity/ G-protein coupled receptor protein signalling pathway, signal transduction	integral to membrane, membrane	embryonic (caecum, retina, kidney); lung tumour
Nfkbia (AI462015, I(Kappa)B(alpha), Nfkbi, nuclear factor of kappa light polyp gene enhancer in B-cell 1)*	Notch signalling pathway, protein binding, negative regulation of myeloid cell differentiation, protein import into nucleus, translocation, regulation of cell proliferation	cytoplasm, cytosol, nucleus	widely expressed
*Nodal (Tg.413d)	TGFb signalling, cytokine activity, growth factor activity, anterior-posterior patterning, cell fate commitment, cell migration, gastrulation, determination of left/right symmetry, positive regulation of cell proliferation, stem cell maintenance	extracellular space	embryonic (epiblast, primitive streak, mesoderm, lateral plate)
Notch3 (AW229011)	Notch signalling pathway, DNA binding, calcium ion binding, receptor activity, transcription factor activity, forebrain development, negative regulation of cell differentiation, negative regulation of neuron differentiation, neuron fate commitment, regulation of development	extracellular space, integral to membrane, membrane, nucleus	embryonic; postnatal (heart); mammary gland tumour
Nphs1 (nephrin)	protein binding, JNK cascade, MAPKKK cascade, cell adhesion	extracellular space, integral to plasma membrane	embryonic (renal cortex, testis, germ cells); postnatal (kidney, cerebellum)
*Pcdh8 (1700080P15Rik, Papc)	calcium ion binding, morphogenesis of embryonic epithelium, somitogenesis	integral to plasma membrane	embryonic (paraxial mesenchyme, tail) postnatal (kidney, spleen, heart, liver, lung, brain, testis)
*Rasd2 (Rhes, TEM2, TEM-2, AU045414, 4930526B11Rik)	GTP binding, nucleotide binding, locomotory behavior, small GTPase mediated signal transduction	intracellular, membrane	embryonic (16-cell stage, brain); postnatal (brain, spleen, colon, lung, testis, salivary gland)
*Rhob (Arh6, Arhb, AA017882,	GTPase activity, nucleotide binding, protein binding, Rho protein signal transduction,	intracellular, membrane,	widely expressed

MGC117867)	angiogenesis, apoptosis, cell adhesion, cell differentiation, development, negative regulation of progression through cell cycle, protein transport, small GTPase mediated signal transductiont	nucleus, plasma membrane	
*Smad7 (Madh7)	TGFb signalling, common-partner SMAD protein phosphorylation, regulation of transcription	intracellular, nucleus	widely expressed
Cripto (Tdgf1, CR1,)	TGFb signalling, activation of MAPK activity, determination of anterior-posterior axis, embryo, positive regulation of cell proliferation	extracellular space, extrinsic to plasma membrane	embryonic (epiblast, pimitive endoderm, ectoderm, mesoderm, primitive streak, heart)postnatal (heart, spleen, brain, lung)

Metabolism

symbol	ontology	component	expression
*GalNAcS-6ST (4631426J05Rik, BRAG, mKIAA0598)	sulfotransferase activity, transferase activity	integral to membrane, membrane	embryonic (visceral endoderm, definitive endoderm, primitive streak, heart, notochord plate, midgut, brain); postnatal (brain, kidney, liver, lung, skin, mammary gland)
Aasdhppt (2010309J24Rik, 2810407B07Rik, AASD-PPT, CGI-80, LYS2, LYS5)	magnesium ion binding, phosphopantetheinyltransferase activity/ fatty acid biosynthesis	cellular component unknown	widely expressed
B3galt3 (B3galt3, Brainiac 1, Globoside blood group, Mbrn 1)	UDP-galactose:beta-N-acetylglucosamine beta-1,3-galactosyltransferase activity, galactosylgalactosylglucosylceramide beta-D-acetylgalactosaminyltransferase activity, galactosyltransferase activity, magnesium ion binding, transferring glycosyl groups/ oligosaccharide biosynthesis, protein amino acid glycosylation	extracellular space, integral to membrane, membrane	widely expressed
*Nxn	antioxidant activity, thioredoxin-disulfide reductase activity, electron transport, cell differentiation,	nucleus, cytoplasm	embryonic (limb, brain, paraxial mesenchyme); postnatal (brain, kidney, liver, lung, skin, heart)
Ppp1r2 (D16Ertd248e, 0610025N14Rik, 2310007G06Rik, 4930440J04Rik)	protein phosphatase inhibitor activity, carbohydrate metabolism, glycogen metabolism, regulation of signal transduction		embryonic (blastocyst); postnatal (eye, head, kidney, mammary gland testis, skin); mammary gland tumour
*Pycr2 (P5cr2, 1810018M05Rik)	oxidoreductase activity, pyrroline-5-carboxylate reductase activity, amino acid biosynthesis, electron transport, proline biosynthesis		widely expressed; mammary gland ad lung tumour
*Slc7a7 (my+lat1, AI790233)	amino acid-polyamine transporter activity, basic amino acid permease activity, carrier activity, amino acid metabolism, amino acid transport, protein complex assembly	integral to plasma membrane	widely expressed; mammary gland ad lung tumour

Apoptosis

symbol	ontology	component	expression
Bbc3 (PUMA, PUMA/JFY1)	protein binding, DNA damage response, induction of apoptosis, negative regulation of cell growth	mitochondrial envelope, mitochondrion	postnatal (kidney, liver, olfactory epithelium); lung and mammary gland tumour
*Mcl1 (AW556805)	protein binding, cell differentiation, development	integral to membrane, membrane, mitochondrion, nucleus	widely expressed
*Moap1 (MAP-1, PNMA4, AA987038,	protein binding	nucleus	embryonic (E7.5, liver);

1700051B17Rik, 2510001G02Rik, 4930435G24Rik, 9130023M10Rik)			postnatal (liver, testis, thymus)
Pea15 (Mat1, PEA-15, Pkcs15)	protein binding, protein kinase C binding, sugar porter activity/ intracellular signalling cascade, regulation of apoptosis, transport	cytosol, membrane fraction	widely expressed

Transport

symbol	ontology	component	expression
*1443256_at (Clca6, AI504701, 9130020L07Rik)	chloride channel activity, ligand-gated ion channel activity/ chloride transport	extracellular matrix, plasma membrane	postnatal (small intestine, caecum)
5730419I09Rik (C030008B15Rik)	transporter activity	membrane, synaptic vesicle	widely expressed
Abcg2 (MXR, ABCP, BCRP, MXR1, ABC15, Bcrp1, AI428558)	ATP binding, ATPase activity, nucleotide binding	integral to membrane, membrane	embryonic (E13.5-15.5); postnatal (hematopoietic progenitor cells, kidney)
Hrb (RAB, Rip, C85612, AU045498, D730048C23Rik)	DNA binding, metal ion binding, zinc ion binding, acrosome formation, cell differentiation, development, regulation of GTPase activity, spermatogenesis	cytoplasmic membrane-bound vesicle, nucleus	embryonic (16-cell stage, blastocyst, E7.5, head); postnatal (testis, spleen, mammary gland); mammary gland tumour

Ubiquitin cycle

symbol	ontology	component	expression
*Ubr7 (5730410I19Rik, AA589405, AW557761)	ubiquitin-protein ligase activity, integrase activity, metal ion binding, protein binding, zinc ion binding, DNA integration, regulation of transcription, DNA-dependent		widely expressed
*Fbxl20 (Scrapper, Fbl2, C86145, AI849362, AL117906, mKIAA4147, 4632423N09Rik)	ubiquitin cycle		widely expressed; mammary gland and lung tumour

Translation

symbol	ontology	component	expression
Eif3s6ip (0610011H21Rik, HSP-66Y, MGC:37328, PAF67)	protein binding, translation initiation factor activity/ response to unfolded protein	fibrillar center, nucleolus, nucleoplasm	widely expressed
Mrpl15 (HSPC145, MRP-L7, Rpml7)	structural constituent of ribosome/ mitochondrial genome maintenance, protein biosynthesis,	intracellular, mitochondrial large ribosomal subunit, mitochondria	embryonic (widely expressed); postnatal (spleen, brain, bone marrow, mammary gland, mammary); gland tumour

Various/Unknown

symbol	ontology	component	expression
*Duxbl (1110051B16Rik, MGC67567)		nucleus	embryonic (E9.0, E18.0 body, brain); postnatal (olfactory bulb)
*Cnpy1 (1500012D20Rik, 9630008K15Rik)			embryonic (2-cell stage, brain); postnatal (cerebellum)
BC037674 (Wapal, FOE, mWAPL, KIAA0261, A530089A20Rik)	response to toxin, spermatogenesis	synaptomenal complex	widely expressed
*D030056L22Rik			widely expressed

Dppa2 (2410088E07Rik)	developmental pluripotency associated 2, nucleic acid binding	nucleus	embryonic (blastocyst, germ cells)
*Plekha2 (TAPP2, W91773, AI851530, AU041791, 6430512N22Rik)	lipid binding, phosphatidylinositol binding	membrane, nucleus	embryonic (4-cell stage, E10.5) postnatal (thymus, olfactory bulb, bone marrow); mammary gland tumour
*Schip1 (Nf2ip, SCHIP-1)	protein binding, protein homodimerization activity	cytoplasm	widely expressed
Sntb2 (Snt2)	actin binding, calcium ion binding, calmodulin binding, protein binding	cytoskeleton, membrane, microtubule, synapse	embryonic (E19.0); postnatal (eye, thymus, diaphragm, vein, hippocampus, aorta); mammary gland tumour
*Tmem63a (Tmem64a, BC014795, MGC11687, MGC25803)		integral to membrane	embryonic (2-cell stage, 8-cell stage); lung and mammary gland tumour
*Tmepai (STAG1, N4wbp4, PMEPA1, AW455466, 2210418I02Rik)	protein binding	integral to membrane, membrane	postnatal (head, colon, ovary); lung tumour, mammary gland tumour
Ttc13 (BC017545, MGC28881, MGC105232)	tetratricopeptide repeat domain 13		widely expressed
